# A novel approach for the detection of brain tumor and its classification via end-to-end vision transformer - CNN architecture

**DOI:** 10.3389/fonc.2025.1508451

**Published:** 2025-03-10

**Authors:** K. Chandraprabha, L. Ganesan, K. Baskaran

**Affiliations:** ^1^ Department of Computer Science and Engineering, Alagappa Chettiar Government College of Engineering and Technology, Karaikudi, Tamil Nadu, India; ^2^ Department of Computer Science and Engineering (CSE), Alagappa Chettiar Government College of Engineering and Technology, Karaikudi, Tamil Nadu, India; ^3^ Department of Electrical and Electronics Engineering (EEE), Alagappa Chettiar Government College of Engineering and Technology, Karaikudi, Tamil Nadu, India

**Keywords:** MRI processing, brain tumor detection, brain tumor classification, end-to-end vision transformer, deep learning, Convolution Neural Network

## Abstract

The diagnosis and treatment of brain tumors can be challenging. They are a main cause of central nervous system disorder and uncontrolled proliferation. Early detection is also very important to ensure that the intervention is successful and delayed diagnosis is a significant factor contributing to lower survival rates for specific types. This is because the doctors lack the necessary experience and expertise to carry out this procedure. Classification systems are required for the detection of brain tumor and Histopathology is a vital part of brain tumor diagnosis. Despite the numerous automated tools that have been used in this field, surgeons still need to manually generate annotations for the areas of interest in the images. The report presents a vision transformer that can analyze brain tumors utilizing the Convolution Neural Network framework. The study’s goal is to create an image that can distinguish malignant tumors in the brain. The experiments are performed on a dataset of 4,855 image featuring various tumor classes. This model is able to achieve a 99.64% accuracy. It has a 95% confidence interval and a 99.42% accuracy rate. The proposed method is more accurate than current computer vision techniques which only aim to achieve an accuracy range between 95% and 98%. The results of our study indicate that the use of the ViT model could lead to better treatment and diagnosis of brain tumors. The models performance is evaluated according to various criteria, such as sensitivity, precision, recall, and specificity. The suggested technique demonstrated superior results over current methods. The research results reinforced the utilization of the ViT model for identifying brain tumors. The information it offers will serve as a basis for further research on this area.

## 1 Introduction

Brain cancer develops within the brain. Symptoms can include memory loss, speech changes, frequent headaches, and difficulty concentrating. Some brain tumors can persist for extended periods (1). Malignant brain tumors, characterized by rapid growth, invasion of surrounding tissues, and indistinct borders, The spread of Brain tumor to other areas of the spinal cord or brain can be considered a serious concern. On the other hand, the growth of benign tumors is slow, and they do not invade other tissues (2).

There are two kinds of brain cancer. These are primary and secondary. The spread of cancer cells from one organ to another can lead to secondary brain tumors. They can occur through the lymphatic or hematogenous pathways. For instance, certain types of cancer are known to migrate to the cerebral region. Lung cancer is a primary source of these tumors. Aggressive forms of breast cancer, such as triple-negative, are known to carry a significant risk of brain metastases. Melanoma, which is known to spread beyond the body, often affects the brain. Colorectal cancer, on the other hand, is less common but can also trigger metastases in the cerebral region. Understanding the various sources of brain tumors can help develop effective diagnostic and therapeutic procedures (3). According to the WHO, a brain tumor can be classified into four phases. It can be classified as a type of cancer. A grade is assigned to brain tumors based on their growth rate, as well as the appearance of abnormal cells under a microscope. Grade I tumors are the least aggressive, while Grade IV tumors are the most aggressive. While staging is not commonly used for brain tumors as it is for other cancers, early detection of the disease and treatment are crucial for improving save lives (4).

Various types of treatment methods are used for brain tumors. These include surgery, chemical treatments, and ultra-violet radiation. Early detection is very important for brain tumors as they can affect the lives of patients and their families (5). The standard method for imaging tumors in the brain is MRI. This method utilizes radio waves and magnetic fields. CT scans use X-rays, while those for detecting brain tumors are made using PET. Radioactive substances are placed in blood to visualize the tumor (6). Biopsy, which involves using a microscope to examine a small portion of the tumor, is the most accurate method of detecting brain tumors. In some cases, conventional imaging techniques are not always capable of detecting brain tumors. These scans can be time-consuming and costly. It can be very challenging for patients with chronic conditions to maintain regular scans (7).

MRI manual extraction involves segmenting or identifying particular regions or structures in the images that were taken using the scanner. A human expert, like a radiologist, can do this. They would draw boundaries or manually trace the structures around them using software. The use of manual extraction for MRI scans can have various drawbacks. This can be time-consuming, labor-intensive, and costly. In addition, it can lead to human error, which can result in inaccurate results (8).

On the other hand, automated systems for extracting MRI data have numerous advantages, such as reducing time and expense, ensuring accuracy, enhancing treatments, and improving the quality of care for patients. These systems do not encounter human error, and they can process vast amounts of data in a quicker than manual approach, rendering manual intervention unnecessary (9). Automated systems can be utilized to optimize the treatment outcome for those with brain tumors, allowing them to receive the most out of their treatment and save their lives (10). In addition, knowing the warning signs and seeking medical help immediately can greatly enhance their chances of survival. Automated systems handle more data than human workers, and their accuracy is better due to how tumors’ size and location affect the various imaging techniques (11).

There are various methods that have been tried to categorize brain tumors utilizing MRI scans. Some of these include utilizing machine learning methods like the KNN, RF, and SVM, which were developed by taking advantage of the unique features of MRI scans. The development of CNN models in 2022 prompted the creation of computational resources that enabled deep learning programs to be created. This led to the creation of new approaches for studying brain tumors. These methods were developed using the existing models. The Inception, Xception, and Visual Geometry Group 16 (VGG16) models are commonly used in computer vision applications (12). The researchers were able to use these models to accurately identify brain tumors, demonstrating the potential of pre-trained computer models. They were correspondingly able to use various other methods for generating synthetic data. In a recent study, the researchers utilized variational auto encoders and adversarial networks for generating synthetic data. They were also able to use ResNet50 for their tumor classification. A study conducted on the Residual Networks (ResNet50), VGG16, VGG19, and Densely Connected Convolutional Networks (DenseNet21) models revealed that the former performed better than the others did. The authors also noted that CNNs tend to have inductive biases, such as the translation equivariance. CNN models performance when evaluating long-range data may be affected by certain biases. They also require data augmentation to improve their performance (13).

The authors utilized different methods to identify and categorize brain tumors. Some of these include the Non-dominated Sorting Genetic Algorithm (NSGA), You Only Look Once version 2 (YOLOv2), Latent Dirichlet allocation (LDA), SVM, KNN inception, and LDA. The algorithm was developed by utilizing the BRATs dataset, which included both LGG and HGG scans. Due to the algorithm’s individual test, its performance is not known for other datasets. A method that can accurately segment and identify brain tumors using MRI scans was presented. The suggested structure for this method is a bitr-unit that comprises of an encoding and decoding component. In spite of the potential of deep learning and AI to identify tumors, they are incapable of being utilized effectively. The accuracy and volume of the data are the factors that affect the output of these algorithms. In some cases, they may not be capable of distinguishing between unusual and uncommon types of tumors (14). Neural networks can be utilized to implement the ViT algorithm, which is designed to recognize images. The algorithm can identify complicated tumors by breaking down the data into parts. For a test, it produces a probability map showing the areas of the brain where the cancer cells are located. Through the use of MRI data and images, trained models can then extract other details. The BraTS collection is one of the most prominent sources of data on brain tumors. It includes MRI scans and images of common types of the disease (15).

Deep learning has been studied for the classification of brain tumors. In a study conducted by Ahmad et al., they were able to achieve an accuracy of 96.25% used a framework that combines an adversarial network and an encoder-decoder network (16). Talukder et al. investigated transfer learning with various architectures like ResNet50V4 and InceptionResNetV2, achieved accuracies up to 99.68% but noted the need for improved image quality (17). Polat et al. also employed transfer learning with VGG, ResNet, and DenseNet models, achieved a top accuracy of 99.02% used DenseNet121 and highlighting the benefits of transfer learning for reduced training time and data requirements (18). Samee et al. developed a hybrid model combining AlexNet and GoogleNet architectures, demonstrating superior accuracy and sensitivity compared to existing transfer learning techniques and traditional machine learning methods (19). Alanazi et al. aimed to analyzed the transfer learning technique’s efficiency in differentiating brain tumors from MRI scans and achieved accuracy (96.9%) on a large dataset and highlighting the model’s potential for real-time applications (20). Ullah et al. compared nine deep learning models, finding InceptionNetv2 to be the most accurate for distinguishing between gliomas, meningiomas, and pituitary tumors. They highlighted the need for larger datasets to improved model performance and reduce training time (21). Mouhafid et al. focused on hyperparameter optimization for brain tumor classification, achieving 98.70% accuracy with an optimized CNN model and emphasizing the importance of hyperparameter tuning for maximizing model performance. They also suggested incorporating a larger and more diverse dataset, including normal brain images, for future research (22).

Despite the promising results, these studies highlight some limitations. Several studies emphasize the need for larger, more diverse datasets to improve model generalization and prevent overfitting (23). Additionally, the computational cost of training complex deep learning models, especially with limited data, is a concern. Finally, further research on optimizing model architectures and hyper parameters is crucial for maximizing performance and adapting models for real-world clinical settings. The author Bonada et al. (41) presened were potential applications of deep learning and AI in analyzing brain tumors. MRI data may be utilized to train such systems, which can then be used to eliminate errors and segment tumors. Deep learning may be utilized to enhance the scanner's accuracy and efficacy in determining and treating brain tumors. Although deep learning can be used in clinical practice, it faces various ethical and practical issues. One of these is the training of models on large MRI datasets, as this will help improve their performance. Further studies are required to address the issues related to the segmentation of post-operative images. New tools that have been developed with deep learning capabilities have the potential to provide valuable therapeutic insights.

Identifying brain tumors precisely from MRI scans is a critical step in improving the treatment and phenomenon for patients. Due to the varying characteristics of brain tumors, image analysis is challenging. This is because the acquisition and image characteristics can vary. In addition to the type and size of the tumor, other factors such as its location and shape can also be taken into account to evaluate the prognosis, other factors such as pixel spacing and contrast can also affect the development of effective anomaly detection techniques. The benchmark dataset is an essential part of any effort to evaluate and compare different techniques for detecting brain tumors. Unfortunately, the variations in the equipment and protocols used in the imaging process can make it difficult to thoroughly analyze the images. The paper presents a novel technique for identifying brain tumors. The development of this method seeks to overcome the challenges of image variability and optimize its diagnosis accuracy.

The use of architectural models to compare and contrast the structures of brain tumors is a step in the right direction, though it also highlights the need to improve the accuracy of diagnosis. Instead of focusing on the main types of brain tumors, the CNN versus ViT analysis should have looked into developing a method that can automatically categorize them namely, Pituitary, Menengioma, and Glioma. The reduction in the time it takes to carry out surgery and diagnose it could be beneficial. The contributions of the work are:

1. In order to properly categorize brain tumors, utilizing ViT-based specimens it does not require specialized training.
2. The enhanced capabilities of ViT models for imaging brain tumors could be achieve through further refinement.
3. A detailed analysis of the ViT models will be organized to determine the appropriate classification standards for brain tumors.

## 2 Materials and methods

### 2.1 Dataset overview

There were 5712 MRI scans of the human brain made available on Kaggle. The repository’s MRI data was used to train and validate the various techniques and models used in this study. The dataset is known as Msoud (33) was a composite of the three aforementioned sources. 1) Figshare (34), 2) SARTAJ (35) and 3) Br35H (36).The scans were categorized into four categories. An MRI-based procedure aims to identify and categorize tumors based on their grade, location, and type. The method is initially carried out by utilizing a single model for every classification task. The malignant tumor known as the Glioma is frequently found in the brain. Another type of tumor is the Meningioma, which can affect the spinal cord area. MRI scans did not find traces of tumors in the brain. The area surrounding the brain’s base is known as the pituitary gland region, and tumors usually appear there. The dataset consists of two folders: testing and training. The first one contains almost 4855 MRI scans. They were used to generate the suggested model. The second dataset included 857 scans, which were utilized to test different assumptions. The data collected from various medical institutions and hospitals has been carefully curated to provide a variety of tumor types and image characteristics. Different protocols and scanners were used to acquire MRI scans, which made the dataset even more diverse. The data included in this collection is marked with binary labels that indicate the presence or absence of a tumor. Experienced medical experts and radiologists ensured the labels’ reliability by performing annotations. The data collected for this project has been subjected to rigorous quality control measures. All images were checked for various issues, such as noise and artifacts.

The information collected from the MRI brain tumor dataset can be applied for a extensive range of applications, including the creation of models for the classification and detection of brain tumors. Healthcare professionals and researchers can utilize Kaggle to access the data. The platform serves as a vital source of knowledge in the area of medical imaging and machine learning. It offers a framework that enables the creation and testing of algorithms that can be utilized in the detection and classification of tumors in the brain. The diverse array of tumor types and image characteristics makes the data set useful for developing models that can generalize to other unlabeled images. The data set is carefully curated and annotated to ensure its usefulness and reliability.

To address the limitations of relying solely on public datasets when training Vision Transformers, several strategies can be employed. One approach is to augment the training data with diverse datasets, including data from different clinical settings, demographics, and imaging modalities. This helps to ensure the model learns a broader representation of tumor variations and diminishes the risk of overfitting to specific characteristics of the public datasets. Transfer learning can be used to enhance the training performance of a model by training it on a larger set of data. This can then be used to fine-tune the model’s classification capabilities. In addition, it can be used to augment the data by implementing various techniques such as color jittering, random cropping, and rotation. These techniques can help reduce the overfitting of the model.

#### 2.1.1 Including and excluding scan criteria

The study seeks to find out if this condition affects the functioning of the brain and what causes developmental delay in individuals with compromised cognitive function who were referred for MRI. They were excluded from the study due to their conditions, such as being infected with diseases such as tonsillitis and pneumonia, or having genetic disorders. The investigation was decided on using neuroimaging due to the limited number of genetic and metabolic tests available and the financial constraints. All children with a delay in development were examined using MRI scans. The sequences used for the study included various types of MRI. Some of these were: the Axial EP2D diffusion, the Axial T2TSE, the Axial PDTSE, the Axial FLAIR, and the Coronal T1TIR. Demographic and clinical details of the enrolled patients were also taken into account.

Meningiomas: Most meningiomas are benign and usually occur in older individuals. They account for around 13 to 25% of all intracerebral tumors.

Gliomas: Most malignant tumors are categorized as gliomas. They account for over 80% of all intracranial malignancies and are known to cause significant morbidity and mortality.

Pituitary Tumors: Most pituitary tumors are benign. They can disturb the body’s hormonal balance and cause severe changes. About 10 to 15 percent of all intracranial tumors are caused by these tumors.

### 2.2 Pre-processing and augmentation of data

Data preprocessing is an integral part of machine learning, as it helps develop models by converting input data into an acceptable format. The research utilizes the torchvision library’ transform.compose() feature to perform various transformations and enhancement procedures. Data augmentation is a vital part of machine learning, as it helps in identifying and classifying images. Data augmentation involves exposing models to varying variations in the input data to help them recognize patterns in various orientations and positions. It can also improve the generalization of the model by minimizing the likelihood that it will deviate from the training data. Moreover, diversifying the training set helps the model obtain a wider variety of learning instances. Noise and variations in the data can be introduced through data augmentation, which can result in overfitting. Machine learning models need to have better robustness and generalization. To achieve it, all input scans should be adjusted to 224*224 pixels. The images should also be rotated and balanced with a probability of 0.5. The value of 0.5 is indicated as the probability that each image will be rotated. On the other hand, with a 50% probability, each image will be horizontal. Randomness can increase the diversity within the dataset and enable the model to learn from different viewpoints. The various parameters of the images, such as their brightness, saturation, and contrast, are adjusted using color jitter. They are then converted into the PyTorch image format using the PIL format. After this, normalization is performed to enhance the model’s training and performance. The random_split() method is used for splitting the data. Preprocessing techniques like augmentation and transformation are then used to boost the model’s efficiency. Volume, quality, and diversity of the collected image data are important factors that can be considered when it comes to improving a machine learning model.

The following steps are utilized to process the collected information.

1. Data Collection and Labeling: We were able to obtain various scans of brain tumors using the Kaggle dataset. These images show numerous types of tumors, such as meningiomas, pituitary tumors, and gliomas.
2. Data Splitting: This ensures the dependability of the model, we split the data with 20% going for testing and 80% for training in Scikit-learn. This method allows us to test the effectiveness of the model on different data sets.
3. Image Resizing: To achieve uniform input dimensions, the images must be adjusted using the target-size parameter in the image data generator in Keras.
4. Color Mode: The utilization of the red, green, and blue (RGB) color scheme in deep learning exercises emphasizes the fundamentals.
5. Label Encoding: A one-hot label encoding procedure is utilized to convert labels into a numerical form. This method can be utilize during training sessions to minimize the loss of categorical information.
6. Data Augmentation: Through data augmentation, we can enhance the model’s generalization and robustness. This process is carried out through the use of various procedures such as rotation, brightness, zoom, and flip. These procedures help improve the learning curve of the model by increasing its diversity.

### 2.3 Overview of deep learning in brain tumors detection

**Figure 1:**
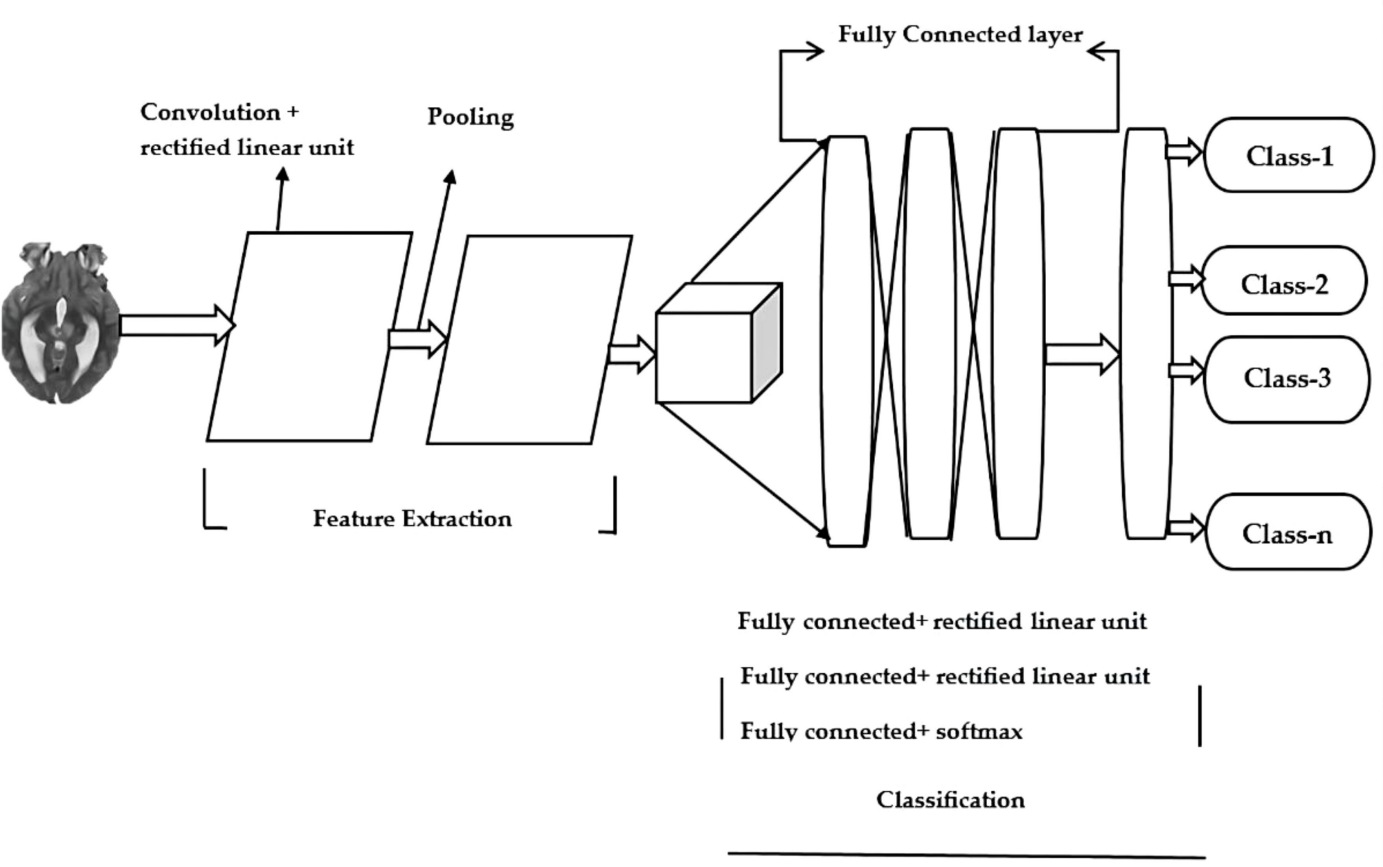
General architecture of Convolution Neural Network.

The field of deep learning focuses on methods that are inspired by neuroscience. In medical image analysis, it can be used to classify and categorize objects. CNNs are frequently utilized in the development of methods for identifying brain tumors. MRI scans can help them learn the relationships among the various voxels in images. A CNN model is made up of different components. Some of these include the Input layer, output layer, pooling layer and activation layer. The latter serves as the gateway that takes the image to the processing network. The various features of an image are extracted using various methods, such as Convolution, pooling and activation. The classification and object detection process is carried out through the connected layer. The outputs of the model are then used by CNN to generate its predictions. The **Figure 1** shows the general architecture.

An MRI scan is used to input data into a deep learning model. The resulting images show the brain in a three-dimensional format, which helps with identifying tumors. In addition to image resizing, other preprocessing techniques are also utilized, such as image normalization and jittering. The training process involves introducing a pretrained model with pooling and convolution layers. The layers used in this process filter the data's features and reduce its dimensionality. A combination of the Dropout, ReLU, and Linear layers can improve the model's overall performance. Prevent fitting and ensure that the data is not distorted. The program outputs a representation of the type of brain tumor that's represented by the class. It takes into account the MRI scan's input and categorizes it into different types. The ReLU6 activation function is a modification of the ReLU linear unit. The ReLU6 reduces the activation size to 6. This helps minimize the precision computations and increases the robustness of the computation and to prevent overfitting. The three additional layers, namely the Linear, Dropout, and ReLU, are also added to improve the performance. The last fully connected layer is then modified to allow multi-classification.

### 2.4 Vision transformers in brain tumor detection

Developers of visual data processing systems, such as those used by computer vision tasks, can now benefit from ViTs, which are advanced deep-learning models. By utilizing a new approach to processing images, ViTs has revolutionized the field. ViTs are different from traditional CNNs in that they handle images as sets of tokens, which enables them to process complex spatial relationships and global information inside images. In the last few years, CNNs have excelled in various vision-related tasks, like segmentation for brain tumors. Because of their small size, CNNs are not capable of handling long-range dependencies. This is because these components depend on the output of distant images. The sequences of medical images are often organized based on the similarities and differences between human organs. CNN models’ performance can be affected by how these sequences are structured. Since these components contain vital information, techniques related to sequence relations can be employed to solve long-range dependencies in images. One of the most common techniques used in developing ViTs is to model the relationships between various token elements. Through this method, the models can learn about global and local feature representations. The method involves modeling the interaction between the different token elements. This enables models that are based on ViT to learn about global and local feature sets.

The vision transformer ViTs uses an encoder-only architecture. It does not have a decoder. Self-attention is used by the researchers to classify images. To segment an image, segment it into smaller patches, which are referred to as patches. These transformations will then turn the patches into tokens, a representation of certain parts of an image is known as a flattened patch. This transition from a two-dimensional to a one-dimensional format allows the model to understand and process the image's various elements. It also helps preserve the relationships and features of the image. Furthermore, the flattened patches become lower-dimensional representations that preserve their crucial features and relationships. Furthermore, additional position embeddings are implemented to retain the information within the structure. The transformations are then sent to the transformer encoder, which allows the model to recognize them as part of the overall structure. The feedforward networks and self-attention layers work together to help the patches learn from one another. The model can also recognize larger patterns and localized features in the image. Unlike traditional methods, which rely on a decoder, ViTs does not have a built-in decoder. Instead, it uses a multi-layer Perceptron known as the MLP head. This enables the model to tackle more intricate tasks, such as object recognition and classification. Self-attention is then utilized to achieve this goal, which makes the ViT an ideal choice for vision applications.

The ViT architecture relies on the class token and the embedding patch projection. The two integral transformations are used in the architecture to provide various features. The patch projection transforms the individual flattened patches into lower-dimensional ones that have distinctive features. This method can be used to extract important details about a particular patch from a model. The class token projection takes advantage of the trainable token vector’s reduction in dimensions to produce a feature vector. The class token serves as a representation of the entire image by merging information from all the patches.

The objective of the Multihead Self Consideration module is to convert the input vectors into key, query, and value vectors, which precede the output's creation. This procedure can aid in identifying the constituent relationships and interdependencies of the image's different elements. Moreover, the module can calculate the sum of the attributes' attention weights. In order to assure the training effectiveness and stability of deep neural networks, various techniques are utilized, such as residual connections and layer normalization.

The ViT architecture features a FFN module. It is a component that can provide a feed-forward neural network. The ReLU activation feature is utilized to convert the output of the module into different feature vectors. This happens through the linear layers.

The following vectors represent the various characteristics of the patch that is featured in the input image. The FFN module can support various layer normalization processes. These stages contribute to the training process’ stability and improve the model’s effectiveness.

The framework is tailored to our requirements by implementing a class structure that was specific to the task. We used the Tensor Flow framework to build a neural network prototype, the initial layer was referred to as the ViT model. The framework for categorizing brain tumors was then improved by adding in task-specific components. The layers were used to update the model and highlight the details in medical images. We also took into account the hyperparameters of the model to ensure that it performs well during training and validation. For training, the model was taught with an Adam optimizer and a learning rate of around 1*10^-4^.

**Figure 2:**
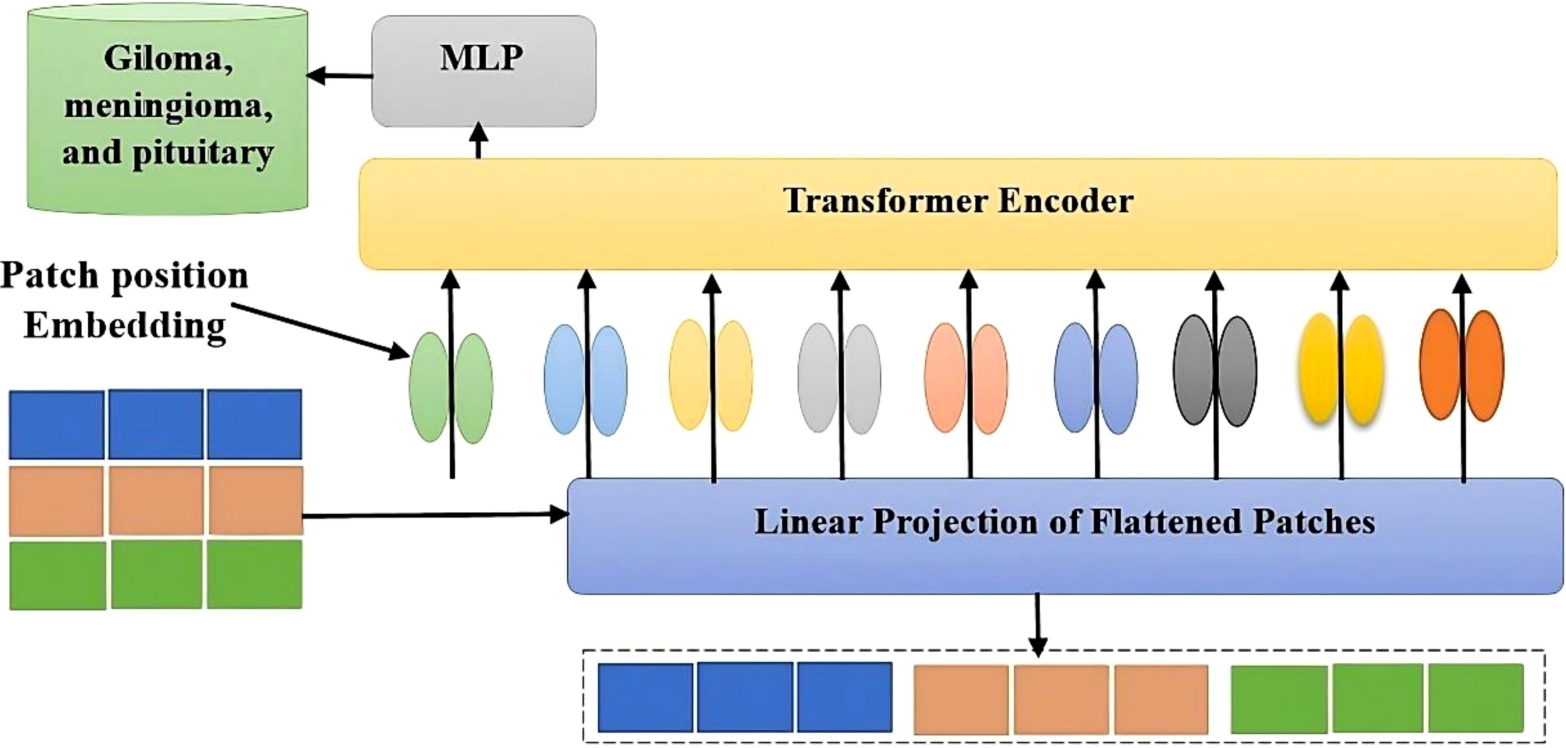
A vision transformer has been used to classify brain tumors based on data.

**Figure 3:**
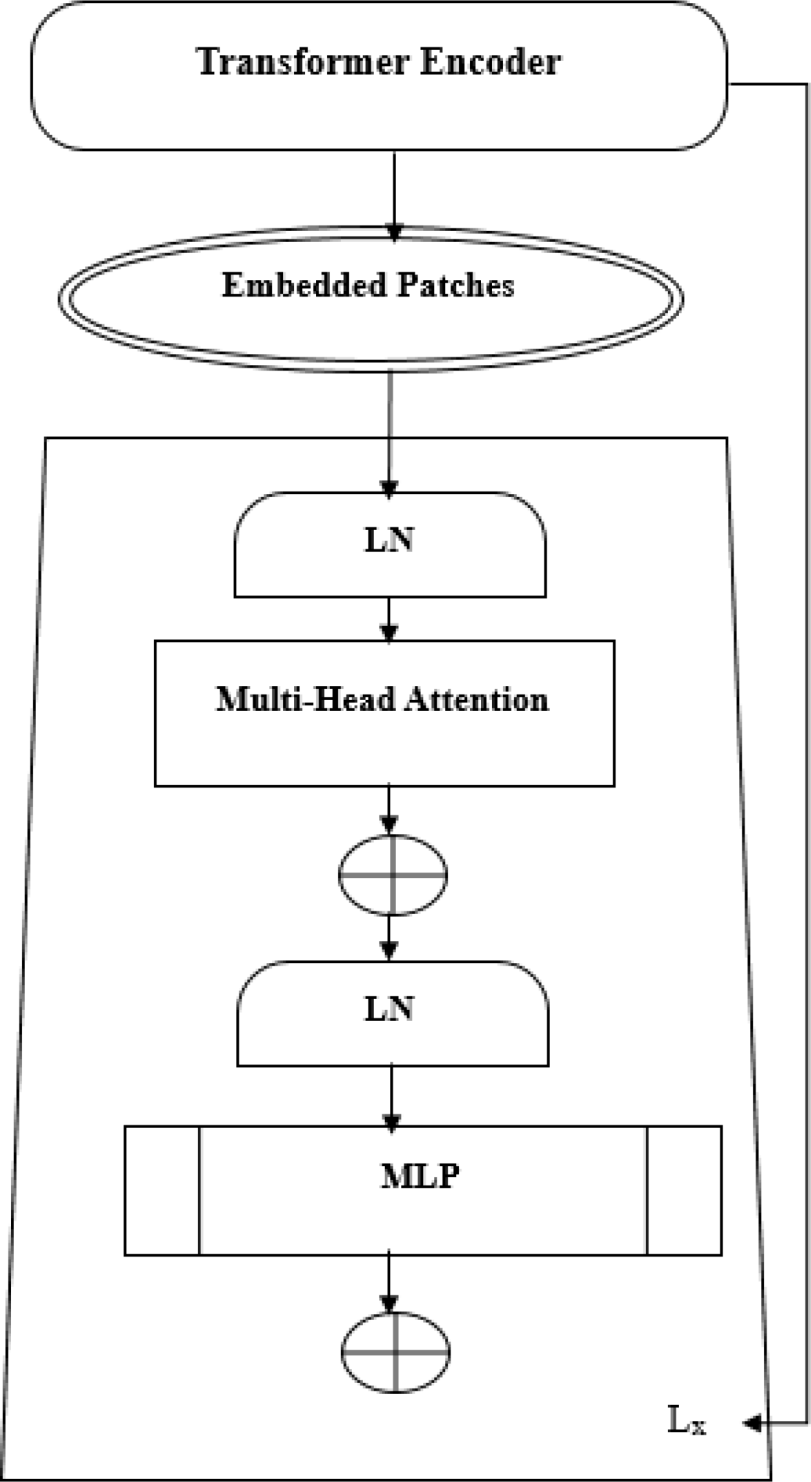
The vision transformer is designed to provide multi-head self-attention.

The ViT model shows its high-level overview in **Figure 2**. In this scheme, the images are arranged into smaller patches, and each of them has n*n pixels. After partitioning the pixels, their values are then flattened. The resulting output sequence is a flattened vector. Flattened patches are then placed into a projection layer, and this produces a linear embedding. Positional alignments are then introduced to the patch to ensure that the information about the image’s position is always available. A transformer encoder is then used to process the embedded sequences and input sequences. The embedded positions are added to the patch’s sequence in order to ensure that they are always updated with the correct data. The input sequences are fed into the transformer. A transformer encoder then processes the embedded and input sequences. A Multilayer Perceptron (MLP) can be used to train the transformer. Multilayered perceptron heads can be used to create new learning patches for the final classification. The positions and embedded patches are then fed into the transformer. It features multi-headed Multilayer Perceptron(MLP) blocks and self-attention in **Figure 3**.

A new learning feature is added to the final classification of a multi-layered perceptron by utilizing a patch embedding. The output of the transformer-encoder model is composed of a set of alternating multi-headed blocks of MLP and self-blocks.

The following are some of the features that are commonly used in the creation of a layer: LN-Layer Normalization, Lx-Transformer En-coder, and MLP-Multi-Layer Perceptron. A learnable embedding is similar to the Bidirectional Encoder Representations from Transformers (BERT) method in that it is composed of the patch embedding sequence. The principle of ViT appears in Equations 1–4. The Epos matrix is the learning parameter in the positional embedding. The first patch N follows a linear projection, while the Z_0_ layer outputs a linear projection. Position embedding can be used to set the order of the patch images. The transformer-encoder layer’s first block begins with a layer normalization. It goes through a multi-headed self-awareness and a residual connection before ending with an MLP. The second block then follows with an MLP and a residual connection to the output Z_1_ and the **Figure 3** shows the transformer encoder model’s MLP. It has two connected Gated Linear Unit(GLU)-nonlinear layers. The output of one of these components is normalized in Equation 4. This indicates that the input image has a final dimension D. The model’s classification head is connected to this output.


(1) $$\begin{array}{c} Z_{0}=[I_{class};x_{p}^{1}E;x_{p}^{2}E;\ldots ;x_{p}^{N}E]+E_{pos}\;E\in {ℜ}^{(P^{2}.c)\times D},E_{pos}\\ \in {ℜ}^{(N+1)\times D} \end{array}$$

(2) $$Z_{l}^{'}=MSA(LN(z_{l-1}))+z_{l-1}\;l=1\ldots L$$

(3) $$Z_{l}=MLP(LN(z_{l}^{'}))+z_{l}^{'}\;l=1\ldots L$$

(4) $$y=LN\;(Z_{L}^{0})$$

The heads in the matrix generate the transformer’s output. In Equation 5, the concept of self-attention is explained as the value, key, and query matrixes are defined after the zl-1 value has been multiplied in Equation 6.


(5) $$H=Attention\;(Q,K,V)=soft\max\left(\frac{QK^{T}}{\sqrt{D}}\right)V$$

(6) $$MultiHead\;(Q,K,V)=Concat\;(head_{1,\ldots ,}head_{h})W^{0}$$

(7) $$head-Attention\;(QW_{i}^{Q},KW_{i}^{k},VW_{i}^{v}$$

The process in Equation 7 helps the model to identify the relationships and dependencies between the various elements of the input sequence. The model takes into account the layer normalization step after the multi-head Attention layer. This procedure ensures that the network’s distribution is consistent. This ensures that the training program’s gradients are stable. This can help improve the model’s generalization and convergence performance.

The model takes into account the extracted features through a MLP, which features two transformations. In Equation 8, a ReLU function separates the transformations. The pipeline’s objective is to extract intricate patterns from the highlighted features. The use of these transformations allows the model to study the data’s representations. For instance, the bias vector of the B2 model is the weight matrix of MLP b1. On the other hand, the W1 of the W2 is the MLP’s weight matrix.


(8) FFNN (x)=ReLU(xW1+b1)W2+b2

The CNN-ViT model employs a customized head that is composed of various components, such as dropout, dense, and ReLU activation layers. This structural change enables the CNN-ViT to perform various tasks specifically based on the collected information. It is more efficient and effective when it comes to adapting to different tasks than tuning methods. The structural modifications allow the CNN-ViT model to take advantage of the data’s features and improve its performance. The structural modifications can help prevent the model from overfitting in small datasets. Also, the inclusion of regularization layers can help prevent it from remembering the noise generated by the training data. The ability to learn from ViT’s learning capabilities can also benefit the CNN-ViT model. This learning method combines the advantages of task-specific finetuning and pre-training. It offers a more efficient way of training.

The ViT framework is built on top of the Conv2d layer. This type of layer is important for enhancing the program’s computational effectiveness. The ViT framework helps minimize the number of parameters that are input in an image before they are processed into the transformer-encoding layers. The framework then passes this information along to the various processing steps, such as patching and embedding. The training batch for the Adam optimizer contains 16 images, which will help the developer learn more about the model. The model’s performance is then measured using the Cross Entropy loss algorithm. Doing so helps the optimizer update the model’s parameters.


(9) $$Adam\;Optimizer\;\theta_{t+1}=\frac{\theta_{t}-\epsilon *m_{t}}{\left(\sqrt{v_{t}}+\eta \right)}$$

In Equation 9, the learning rate is the amount of weight that the Adam optimizer updates during each step of training. The terms vt and m_t_ are used to track the past changes that the optimizer makes in order to improve its efficiency.


(10) $$Cross\;Entropy\;Loss=-1*\sum_{i}^{N}yi*\log(\hat{y}i)$$

In training, this Equation 10 is utilized to determine the distance between the predicted labels and the actual ones. It takes into account various factors, such as the number of samples and the predicted probabilities. For the training phase, the collected data is evaluated and trained using a set of 10 epochs. The head of the classifier is then substituted with a set of custom layers, such as the dropout, BN, and ReLU activation layers. The model is now a framework for training CNN-ViT labels.

The process of normalizing the activations in each batch takes place through the use of the layer known as the BN layer presented in Equation 11. This ensures that the stability and training speed are improved.


(11) $$BN(x)=\frac{\left(x-\mu \right)}{\sqrt{\sigma^{2}}+\epsilon }$$

This layer uses ReLU activation to learn complex relationship structures. Equation 12 can maintain a positive value while turning a negative one to zero.


(12) RELU(x)−max(0,x)

The regularization process of the dropout layer arbitrarily drops neurons to shut out overfitting presented in Equation 13. This prevents the activation of zero during training.


(13) Dropout=x or 0

Linear transformations can be performed with the help of this layer (Equation 14), which takes advantage of the effects of the input vectors’ biases and weights.


(14) Linear(x,W,b)=W∗x+b

The ability to learn more complicated patterns will be enhanced by adding these layers. The size of the final layer has also been adjusted to correspond with the count of brain tumor groups. The output from this part can help in identifying the different kinds of tumors. Which is represented in Equation 15 as follows:


(15) X'=ReLU(BatchNorm(W2∗Dropout(ReLU(BatchNorm(W1∗Z)))))

The above Equation 15 describes the classification head that has been modified. It takes into account the final layers that are responsible for the classification process. The input feature vector for X is 764,024 dimensions. The weight matrices for the linear layers are W1, W2, and BatchNorm. The Rectified linear unit activation function is also called ReLU.

The algorithm’s final output is achieved by passing the system’s cumulative heads through a linear layer. This method is useful in calculating the learning curve for each head. Details about the ViT models’ fine-tuning are also provided in this research. The framework Tensor Flow is used to construct a neural network framework. The initial layer is modeled using the ViT training algorithm. We added task-specific layers to improve the classification of brain tumors. These are then use to regularize the framework and enable it to recognize different patterns in medical images. The classification task is carried out according to a multi-class structure. The loss metric is derived from the sparse categorical, and the performance metric is derived from accuracy. The accuracy and confusion matrix are used during the training and validation stages. The sensitivity of ensembles and their specificity are then evaluated using per-class sensitivity and sensitivities. The tuning model employs various hyper parameters. These include the learning rate, the number of epochs in a cluster, the mini-batch sizes and the Adam optimizer’s maximum size. The optimization of the hyper parameters is carried out through the validation set. The performance metrics are then calculated after the cross-validation procedure. We performed batch size optimizations and learning rate enhancements to ensure that the model would converge and generalize.

## 3 Results

In ViTs, an input image is processed using a patch generator, which is similar to the word token for Natural language processing (NLP) transformers. This process then produces embedded images using the transform encoder. The three components of the transformer encoder block are all related to this process. A vision transformer is a type of image recognition system. It can be utilized for various applications, like object recognition. It is based on the transformer architecture utilized in NLP, which converts text into sequences and generates embedded text.

### 3.1 Dataset exploration

The Kaggle brain tumor MRI dataset consists of 5712 scans of human brains. There are four classes of brain tumors, and the origin of one of them is known as a glial cell. Another type is known as a meningioma, which grows in the membranes surrounding the spinal cord and brain. A magnetic resonance imaging scan did not find a tumor. On the contrary, a tumor can grow in the pituitary gland. The classification of the dataset revealed in **Table 1** that it had two folders: testing and training. The former holds 857 scans that proved various assumptions, while the latter holds 4,855 scans that were utilized to develop a suggested model.

**Table 1:** Summarized results of proposed experiments on train and test data.

Classification types	Image count	Trained data	Tested data
Type-1 (glioma)	1321	1100	221
Type-2 (meningioma)	1339	1144	195
Type-3 (No tumor)	1595	1241	216
Type-4 (Pituitary)	1457	1370	225
Total	5712	4855	857

The experiments that we performed on Kaggle were carried out using the platform’s computational resources. We were able to store and manage our data using the 73.1 GB of disk space that was allocated to us. The Kaggle environment provided us with 13 GB of RAM, which was very important for performing various tasks, such as loading and manipulating data. We also had access to a powerful GPU, which was able to provide us with an efficient and quick boost to the training of deep learning models. In addition to RAM, Kaggle also allocated 19.5 GB of storage for output storage. This was very important for us as it allowed us to store and analyze the various data generated by the experiments.

### 3.2 Data pre-processing and augmentation

**Figure 4:**
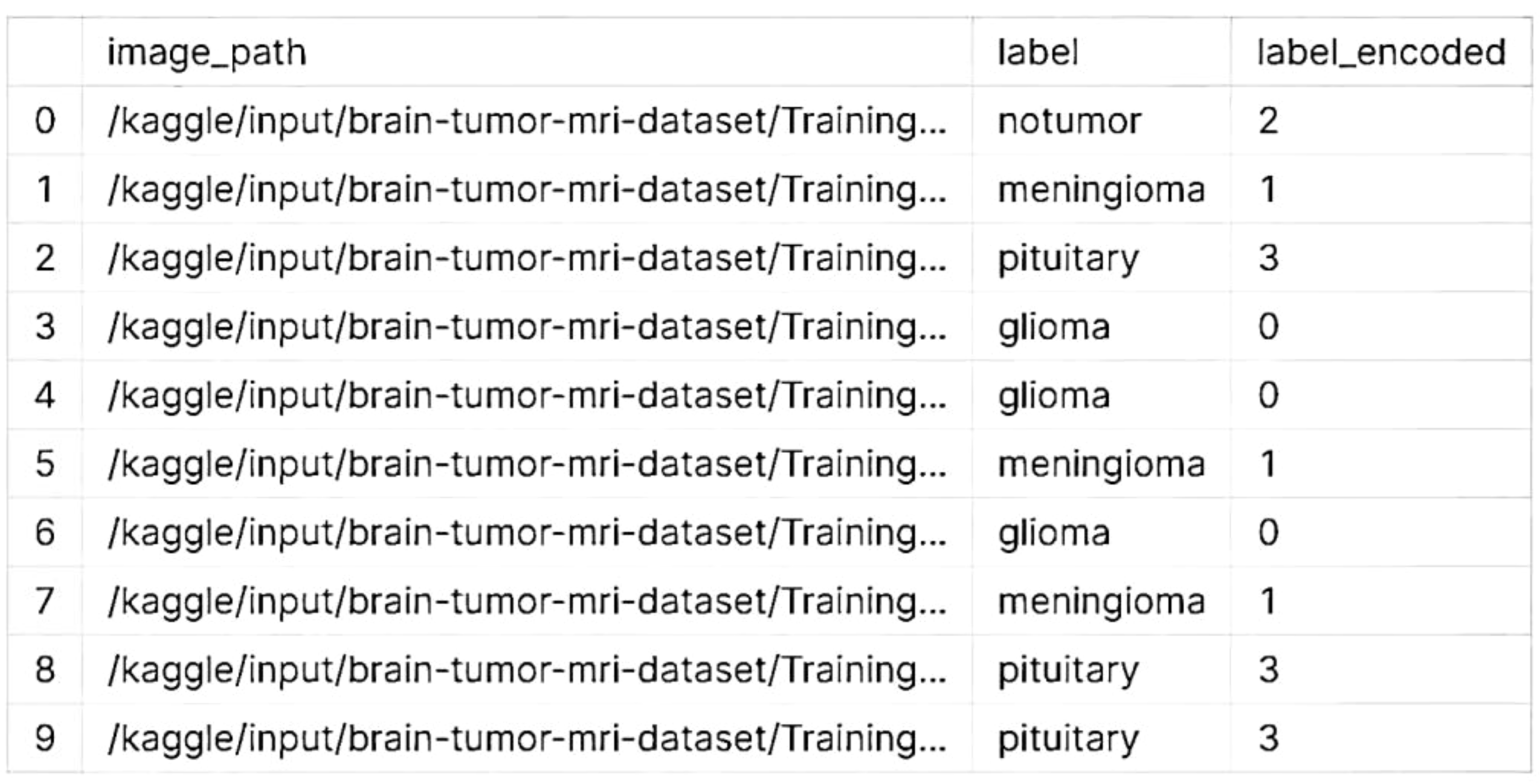
Label encoded classes for first 10 samples.

When processing data related to brain tumors, the classes that are encoded are typically categorized into various types or categories. To make them easier to understand, the labels or names of these classes can be changed. For instance, if the encoded classes are composed of numbers, the first thing that you would do is change the name of one of them to “glioma,” followed by “meningioma,” and finally “pituitary adenoma” is showed in the **Figure 4**.

#### 3.2.1 Create train and test splits

Training and testing a model using a dataset that has 44 classes, like brain tumor classification, usually involves splitting it into two groups. The train_test_split utility will split your dataset into two, with 20% going for testing and 80% for training. Training and testing can be adjusted. The data collected from your brain tumor will be split into two sets, one for testing and one for training. The classes that are represented in the training set are those that are related to the tumor.

#### 3.2.2 Create validation and test splits

We can create test and validation splits for the brain tumor image dataset, as well as training splits. These will be divided into three groups: validation, training, and testing. We first split the data into two groups: a training set and a temporary set. We then split the latter into two sets: one for testing and one for validation. In the second call to the training_test_split, set the size of the validation to match that of the temporary set.

#### 3.2.3 Create an image data augmentation layer

An image data augmentation layer for brain tumor images needs to be created by implementing various transformations. These transformations can be performed on the dataset by altering its brightness, rotation, contrast adjustments, zoom, and more. We replace the labels y_train and x_train with the actual training data. Additionally, adjust the augmentation parameters as needed based on your dataset characteristics.

### 3.3 Modeling using CNN and vision transformers

A CNN is a type of deep learning algorithm that utilizes a grid-like structure to process information. It is mainly used to handle time and space-related data. Although they are similar to other networks, CNNs use a variety of convolutional layers to increase its complexity. Similar to neural networks, CNNs utilize a variety of convolutional layers. As a result, they have an added complexity. One of the most essential components of a CNN is a convolutional layer. The diagram below shows a CNN brain tumor architecture, which is used to predict brain tumors. The objective of this project is to create a CNN that can handle various tasks, and improve its accuracy in predicting the data that it collects.

The Vision Transformers and CNNs have their own architectural differences. CNNs can still achieve remarkable results even when using training data that is not as large as what Vision Transformers require. The CNNs’ inclusion of certain inductive biases may explain the difference between their behavior and that of the Vision Transformers. On the one hand, these networks tend to limit the scope of the analysis they provide, making it more difficult to grasp global relations.

The Vision Transformers are able to capture various global and diverse relations without the need for bias training, which is very beneficial for them. However, it can also be very expensive to obtain the necessary data. The ability of vision transformers to overcome various image distortions, such as permutations and adversarial patches, is a remarkable achievement. In spite of that, choosing the right architecture for a Computer Vision task is not always the best choice. Hybrid architectures, which combine the features of Vision Transformers and convolutional layers, are often successful. **Table 2** shows the architecture layer used in our proposed model prediction.

**Table 2:** Architecture layer used in our proposed model prediction.

Layer	Input	Shape	Parameter
	Output		
Input Layer	input	([None,240,240,3)]	
	output	([None,240,240,3)]	
Conv2D	input	([None,240,240,3)]	896
	output	(None, 119, 119, 32)	
MaxPooling2D	input	([None,119,119,32)]	0
	output	(None, 59, 59, 32)	
Conv2D	input	([None,59,59,32)]	9248
	output	(None, 29, 29, 32)	
MaxPooling2	input	([None,29,29,32)]	0
	output	(None, 14, 14, 32)	
Flatten	input	([None,14,14,32)]	0
	output	(None, 6272)	
Dense	input	([None,6272)]	401472
	output	(None, 64)	
Dropout	input	([None,64)]	0
	output	(None, 64)	
Dense	input	([None,64)]	65
	output	(None, 1)	

The accuracy metric is used by machine learning experts to assess the capabilities of their models to distinguish benign brain images from those with tumors. The accuracy metric is used by engineers to evaluate the predicted output of a project. On the other hand, the loss metric is focused on the difference between the actual value and the desired outcome. Measuring the errors that a model encounters in its input sessions is done with the accuracy metric. It can be used to scrutinize its performance. **Table 3** shows the proposed model with different parameters.

**Table 3:** Proposed model with different parameters.

Model	Patch Size	Backbone	Hidden Units	Accuracy
R50-ViT-l16	16 * 16	ResNet-50	2048	92.50%
Proposed	16 * 16	CNN	2048	99.64%

A model’s validation accuracy and training levels are a good indication of its proficiency. However, when the training accuracy exceeds the validation accuracy, this suggests that the model may be over fitted. When a model is too focused on the details of training data, it cannot efficiently generalize to new data. Reducing the model’s complexity or adding more training data can also help. Low validation and training accuracy values indicate underfitting. Underfitting can occur when a model does not have the necessary complexity to extract the underlying patterns from the data. In these cases, it is beneficial to adopt an ensemble strategy or enhance the model’s complexity.

**Figure 5:**
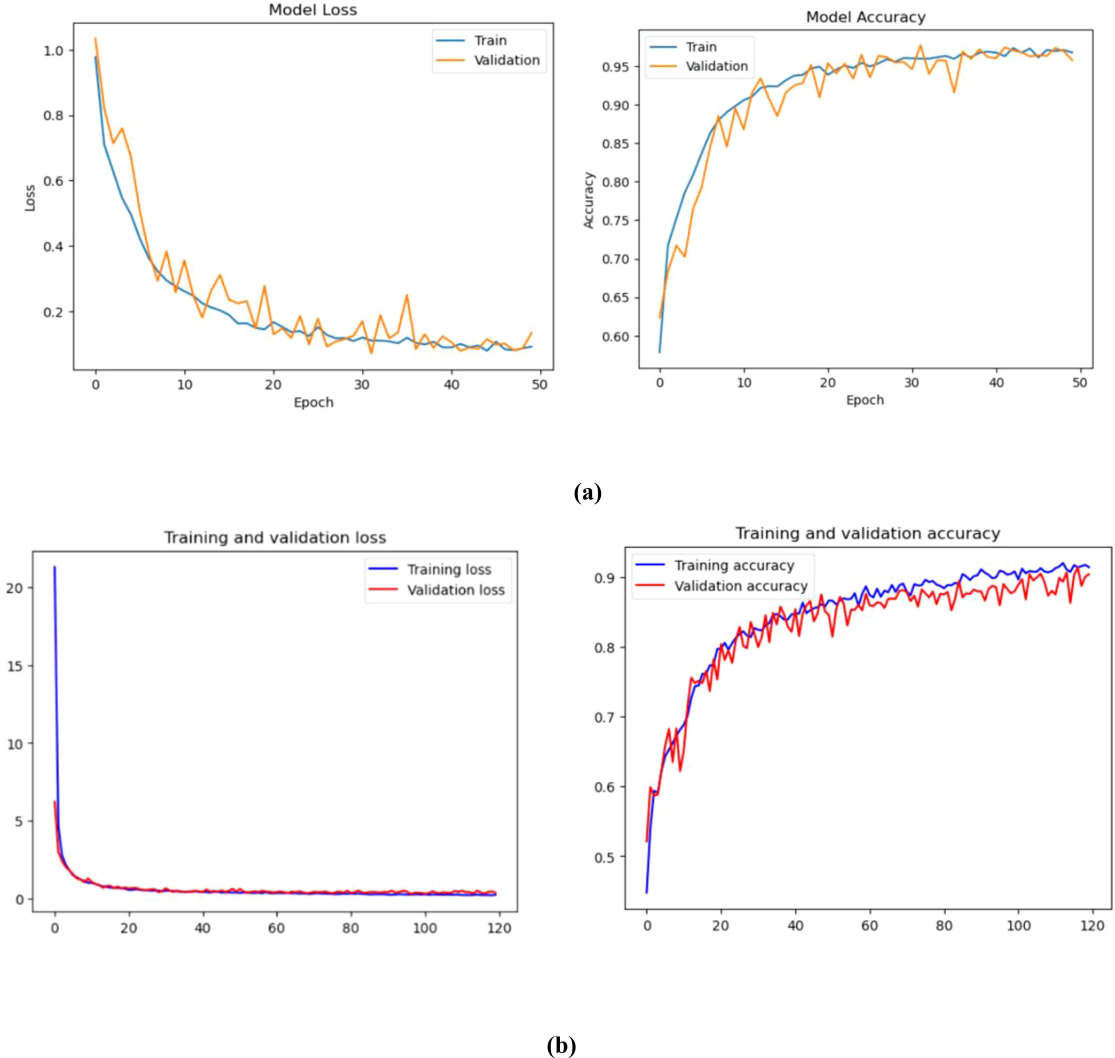
Training and validation loss and accuracy (%) of **(A)** Convolution Neural Network, **(B)** Vision Transformer.

The training of the model is carried out in 50 successive epochs using an adam optimizer. The validation and training accuracy curves gradually improve as each interval passes. **Figure 5** shows the progress of these curves. On the 15th epoch, our model achieves 97.50% and 96.75% accuracy, respectively.

The training and validation graphs are made up of ten epochs. The former displays the training values, while the latter shows the validation indicators. The training accuracy of different models increases as time goes by. This is because their methods are being refined to predict the labels of the data. This phenomenon tends to enhance the training accuracy of the models as time goes on. CNN could indicate that the models are being over-fitted with the data. The training loss associated with proposed models can decrease as epochs increase. This is because the enhanced training capabilities enable the models to fit the data. Unfortunately, the validation loss can also decrease as the number of records goes up. This suggests that the models are overfitting.

**Figure 6:**
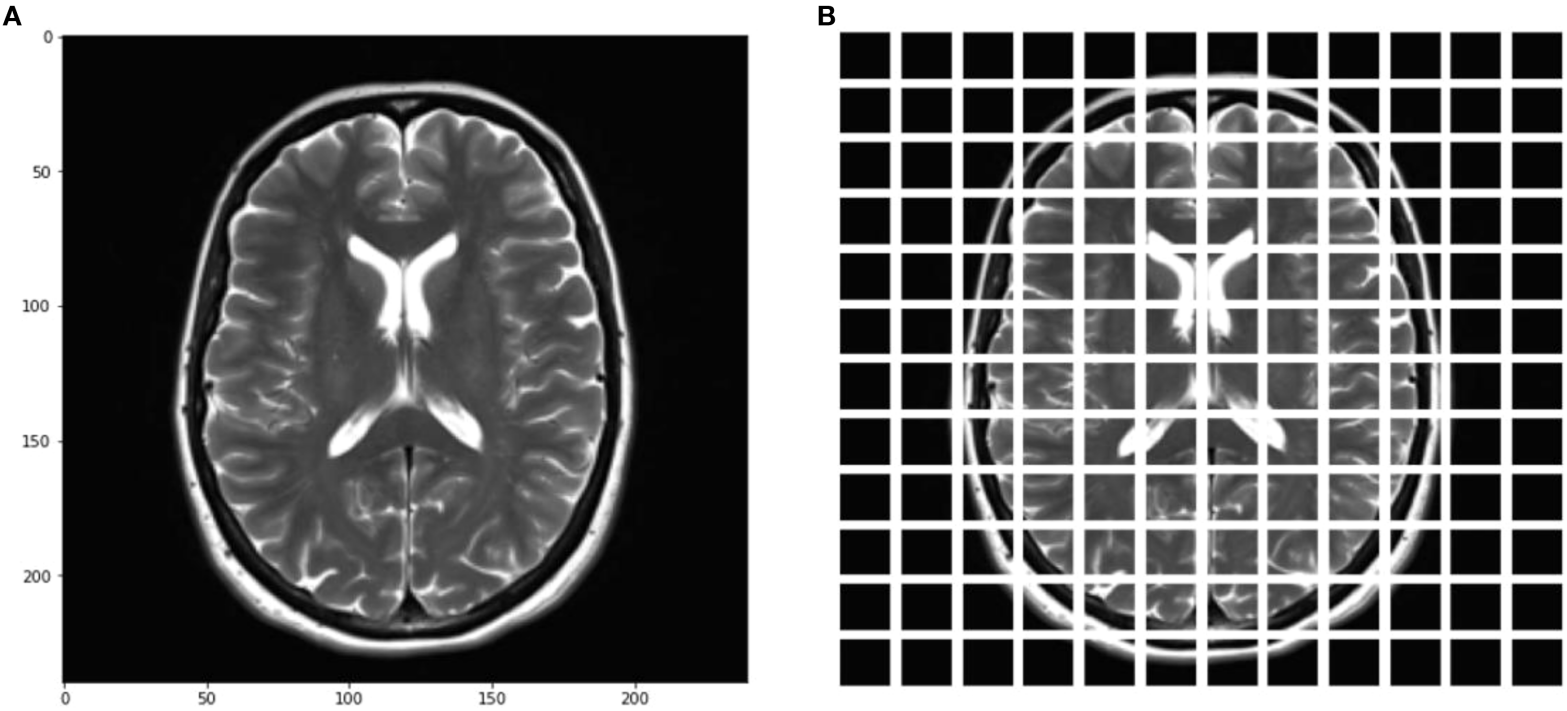
**(A)** Input brain tumor blocks, **(B)** Projection of flattened patches.

**Figure 6** shows the corresponding projection of flattened patches. The patch engraver layer Patch Encoder can transform a patch into a vector with a size projection_dim. It can also add a learnable position that can be embedded in the image. The Vision Transformer architecture is used in this work to address the issue of image manipulation. Unlike other methods, it does not alter the data and does not leave the algorithm untouched. In **Figure 6**, the model shows how it uses the same kind of transformer block as in NLP. The main difference between the two is how they interact with inputs and outputs, and the way they are created. For instance, the Transformer’s input is generated by splitting the image into smaller patches, which then flattens them. This produces a set of vectors that are similar to sentence-like structures. This method combines the generated vectors with positional embeddings. It is similar to how sentences are ordered. Creating embeddings that can encode a patch’s two-dimensional location did not improve the performance. The ViT architecture does not have a decoding block since the model’s latent space vector is big enough to be used for classification. Its outputs are sent to an MLP network, which then makes a decision based on the input. However, instead of a regular input, the architecture uses a learnable class that is similar to the BERT token. The Vision Transformer token is designed to represent the image in a single vector. It can be used alone to make decisions without requiring further outputs from the previous blocks. Its encoder can also perform other tasks, such as extracting features from the image. The difference between the ViT and a CNN is that the former has a lower inductive bias. This means that it can train more correctly, but it also requires more data to extract global connections. The ViT framework is composed of various transformer blocks. Its Multi-Head Attention feature can help address the patches in sequence. The output of these transformer blocks is composed of various parameters, such as the projection size, batch size, and the num-patches tensor. They are processed using a classifier head.

This paper presents a method that enables a learner of embedded patches to create an image representation. The output of the Transformer Block is then adjusted with layers. This transforms the output into a classifier head. The paper suggests using the layers.GlobalPooling1D variable to aggregate the output of the transformer block, particularly when there are several patches and the projection sizes are large.

The paper’s results were achieved through the training of the ViT model on the JFT-300M dataset. The model was then refined to the desired dataset. Without pre-training, the model’s quality can be improved by training it for several epochs. It can also be improved by using more Transformer layers, changing the input images’ size, and increasing the projection dimensions. The ViT model’s quality can be affected by a variety of factors, such as its learning rate, weight decay, and optimizer. In order to improve its performance, it is recommended that one use a high-quality data set.

### 3.4 Performance metrics

The performance of deep learning and machine learning techniques was evaluated using various metrics. Most studies concentrating on the segmentation of brain tumors. There have been many studies that utilized various factors such as precision, sensitivity, specificity, and accuracy to classify brain tumors.

#### 3.4.1 Confusion matrix

**Figure 7:**
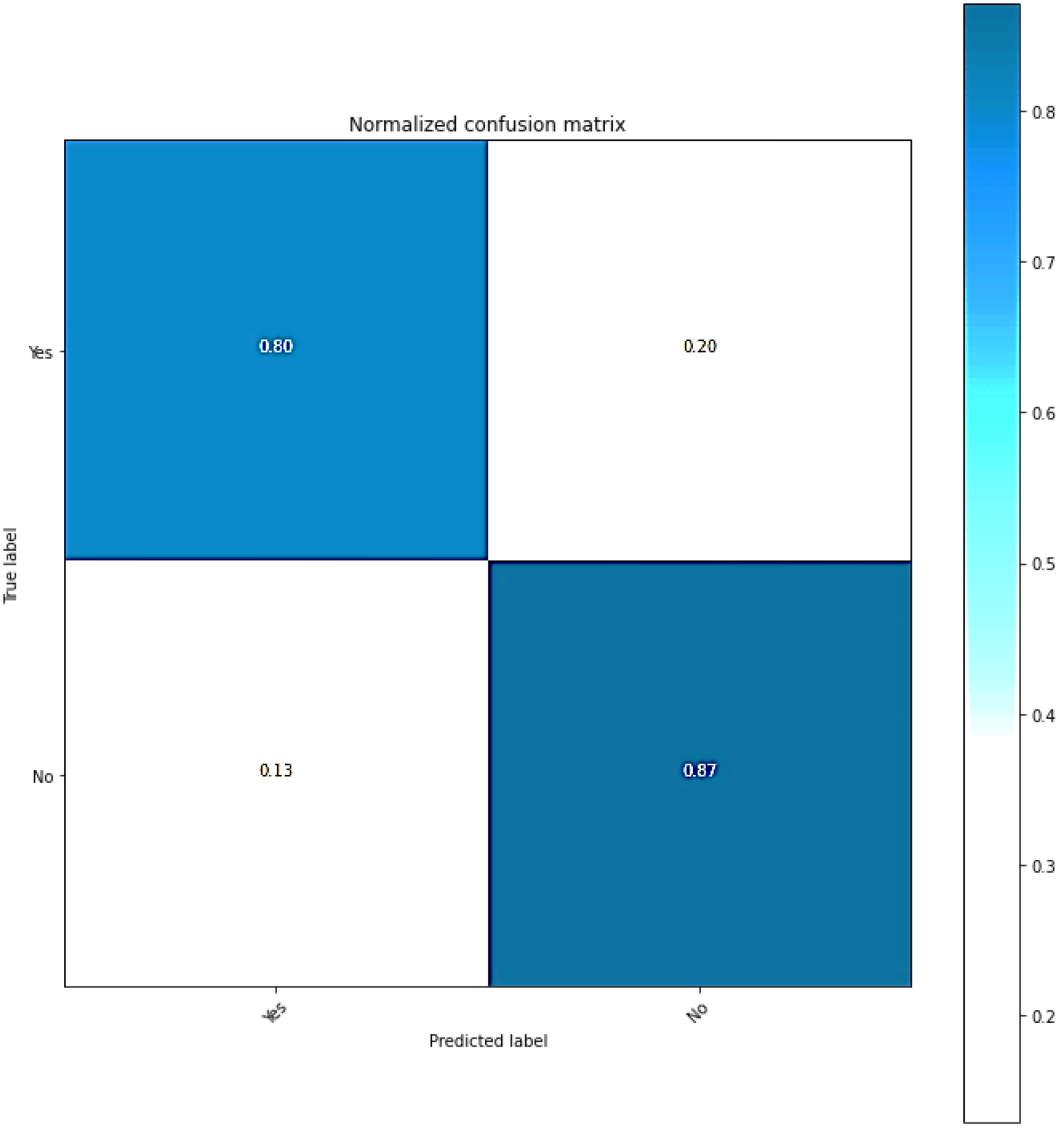
A confusion matrix corresponding to proposed method.

Classification models’ efficacy can be evaluated by utilizing a confusion matrix, wherein the predictions are presented in a tabular format. The **Figure 7** shows the classification model produces error estimates and accuracy ratings for each class. It displays the various combinations of predicted and actual labels in its confusion matrix. The numerical values represent the instances that fall under the given category.

#### 3.4.2 Accuracy

The accuracy (ACC) of a diagnosis or prediction of a brain tumor can be computed using the same standard procedure as in predictive modeling. In the prediction of brain tumors, the model makes various predictions that can represent different outcomes. For instance, the true positives and negatives of brain tumors can vary.

These terms are utilized in the identification and prognosis of brain tumors.

1. True Positive(TP), The correct prediction of a brain tumor was made by a model known as the True Positive.
2. The True Negative(TN), on the other hand, correctly predicted that there would be no brain tumor.
3. The False Positive(FP), on the other hand, incorrectly identified a brain tumor when it was not present.
4. The False Negative(FN), on the other hand, mistakenly states that a brain tumor will not exist if it is present.

With these definitions, the accuracy (ACC) formula for brain tumor prediction would be as follows in Equation 16:


(16) $$\text{Accuracy}=\frac{TP+TN}{TP+TN+FP+FN}$$

The formula takes into account the proportion of the predicted cases that were correct, both positive and negative. Although accuracy can provide a good approximation of a model’s performance, it does not mean that the model is perfect. For instance, if the dataset is imbalanced, its performance might be affected by various factors. In addition to accuracy, it is also important to examine other metrics when performing a comprehensive evaluation.

#### 3.4.3 Precision

The precision (PR) of a model’s positive predictions is computed by taking into account the proportion of accurate predictions as in Equation 17.


(17) $$\text{Precision}=\frac{TP}{TP+FP}$$

#### 3.4.4 Recall

The Recall (RC) metric takes into interpret the number of favorable outcomes that the model can achieve through every favorable case. Equation 18 shows the model’s proficiency in identifying such cases.


(18) $$\text{Recall}=\frac{TP}{TP+FN}$$

#### 3.4.5 F1-score

The concept of the F1-score refers to the harmony between precision and recall presented in Equation 19. It helps to understand a model’s performance when these components are balanced.


(19) F−Score=2∗Precision∗RecallPrecision+Recall

**Figure 8:**
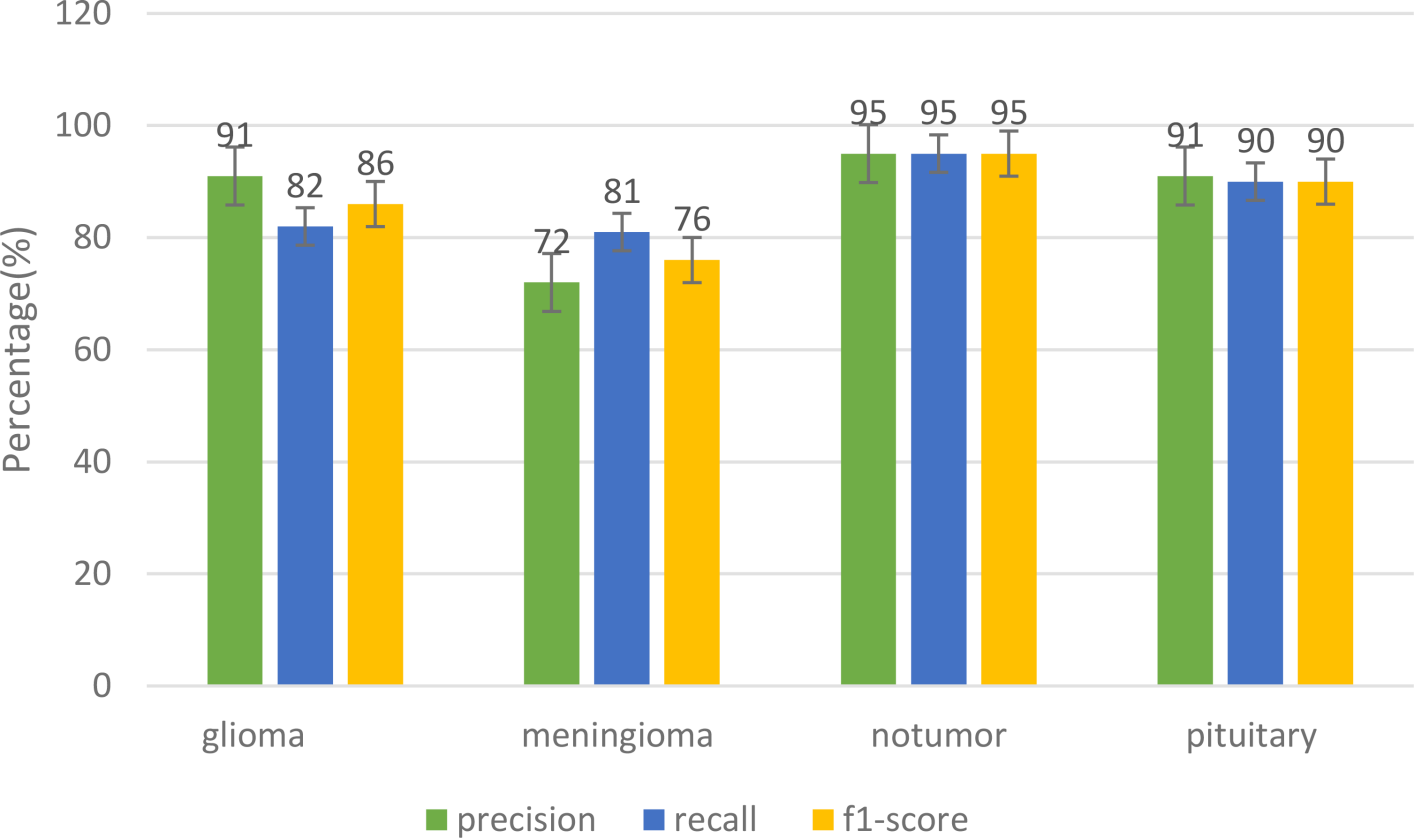
Performance comparison for different tumor categories.

The **Figure 8** shows the various metrics that are used to evaluate the performance of different models in terms of their classification of tumors. The suggested model performed well in the precision category. It had a Precision of 0.91, while its recall score was 0.82, and its f1 score was 0.86. The precision for the Meningioma tumor was at 0.72, while the recall score was at 0.81, and the F1 score was at 0.86. The suggested model was also able to achieve a remarkable 97.89% accuracy. It was able to classify the Glioma tumor with a precision of 0.97, a recall of 0.98, and an F1 score of 0.98. It was also able to perform well in the classification of Pituitary tumors with a precision of 0.91. It was able to achieve an exceptional precision of 0.91 in the classification of the Pituitary tumors. It also performed well in recall with a score of 0.90 and an F1 rating of 0.90. In the classification of three different tumors, the proposed model excelled in its categories, particularly in the precision and recall of No Tumors. It was able to achieve an F1 score of 0.95.

**Figure 9:**
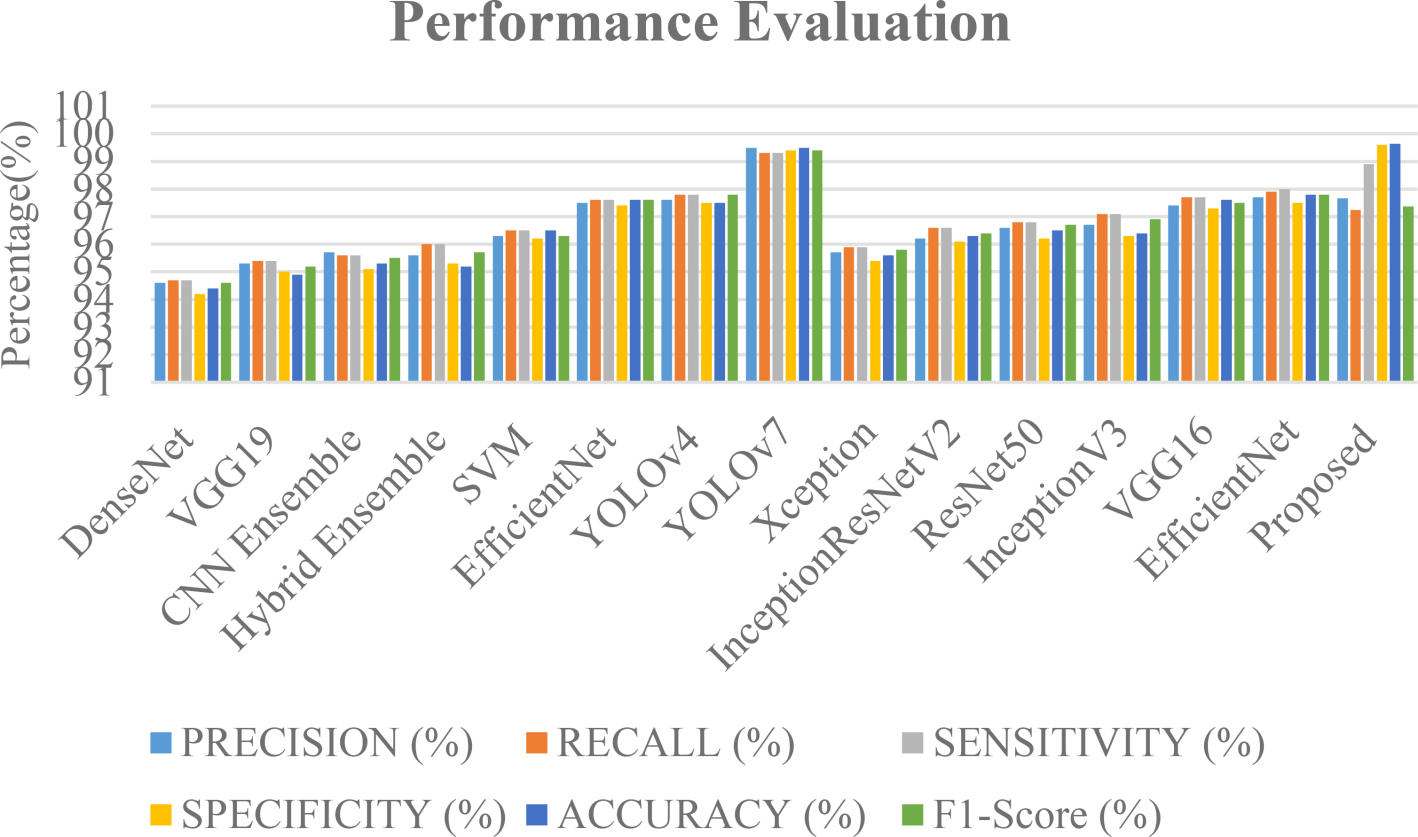
A comparison graph of precision, recall, sensitivity, specificity and F1-score with different approaches.

**Figure 10:**
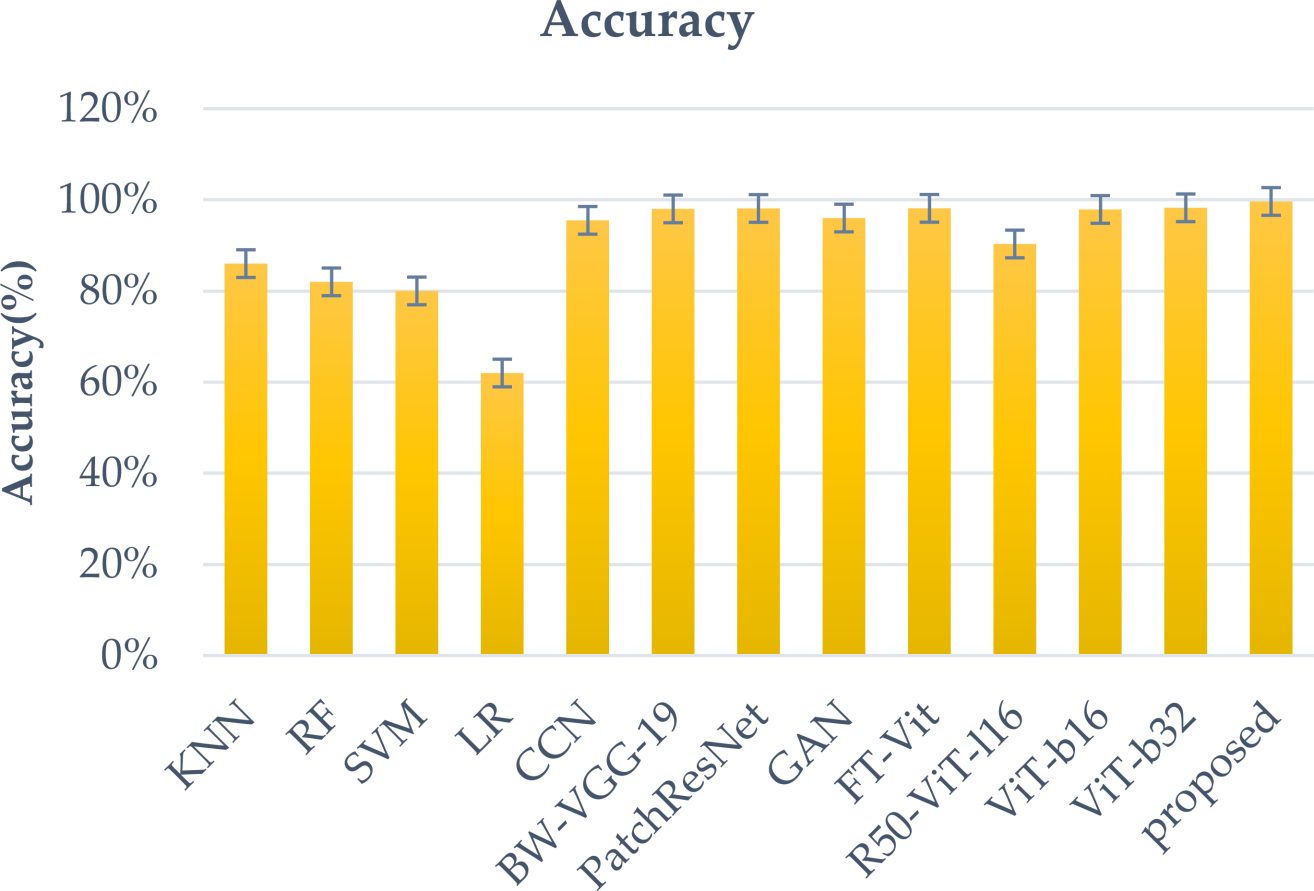
Comparison graph with the existing work.

The **Figure 9** shows the various deep learning models that are likely to be used in a classification task. They are based on various metrics such as recall, precision, sensitivity, specificity, and accuracy. The “Proposed” model boasts the highest accuracy (99.64%) and an exceptional specificity (99.6%), indicating its strength in correctly identifying negative tumor cases. YOLOv7 also demonstrates near-perfect scores across all metrics, achieving 99.5% accuracy. Other high-performing models, such as EfficientNet, YOLOv4, and VGG16, achieve scores in the mid-to-high 90s range, demonstrating their effectiveness. Ensemble methods (45, 46) and SVM (44) also show competitive performance, highlighting the potential of combining models and the continued relevance of traditional machine learning techniques. While DenseNet (42) and VGG19 (43) achieve respectable scores, they lag behind the leading models. For a more comprehensive evaluation and comparison. The data used, as well as the task and proposed methodology, are crucial. An in-depth comprehension of the model’s training strategies, architecture, and novel contributions can be achieved through this study. enabling a more thorough assessment of its advancements over existing methods. The accuracy rates of these techniques are shown in the **Figure 10**.

The goal of this study is to improve the accuracy and clinical relevance of brain tumor classification using preprocessing and fine-tuning of models. It also deploys models on unseen data to analyze and improve the performance of each class. **Figure 10** illustrates a comparative analysis of brain tumor classification accuracy achieved by various Convolutional Neural Network architectures. K-nearest neighbor (KNN), Random Forest (RF), support vector machine (SVM), Linear regression(LR), block-wise-Visual Geometry Group-19(BW-VGG-19), Patch Residual neural network, Generative Adversarial Network(GAN) and Fine-Tuned Vision Transformer (FT-Vit).The results demonstrate a range of performance across different CNN models, with standard CNNs achieving 95.49% accuracy (24). Architectures incorporating domain-specific modifications, such as block-wise-Visual Geometry Group-19(BW-VGG-19) (25) and PatchResNet (26), yielded improved accuracies of 98% and 98.10% respectively. The integration of Generative Adversarial Networks (27) further enhanced performance, reaching 96% accuracy. Vision Transformer based approaches, including Fine-Tuned Vision Transformer (FT-Vit) (28), R50-ViT-l16 and ViT-32 (29), demonstrated competitive results, ranging from 90.31% to 98.254%. The study (30) revealed that K-nearest neighbor (KNN) or support vector machine (SVM) improved the accuracy of the softmax classifier when it came to detecting meningiomas. The sensitivity of the model to overfitting and limited training data highlight its disadvantages. Another study (31) revealed that the KNN or SVM could achieve a 98.7% accuracy rate without segmentation, which is better than the transfer learning methods. However, segmentation still requires a considerable amount of training time. Significantly, the proposed methodology outperformed all other models, achieving the highest accuracy of 99.64%. This suggests that the specific architectural innovations and training strategies employed in the proposed methodology contribute to its superior performance in accurately classifying brain tumors. Further investigation into these novel aspects could provide valuable insights for advancing the field of medical image analysis. The proposed models demonstrate superior precision when it comes to distinguishing between different types of tumors, such as meningioma, glioma, and pituitary. The proposed models’ superior accuracy can be attributed to the use of a custom classifier head, which allows for direct representations of the data. In addition, the suggested models employ attention strategies to enable them to extract intricate patterns and correlations from medical imaging data.

**Figure 11:**
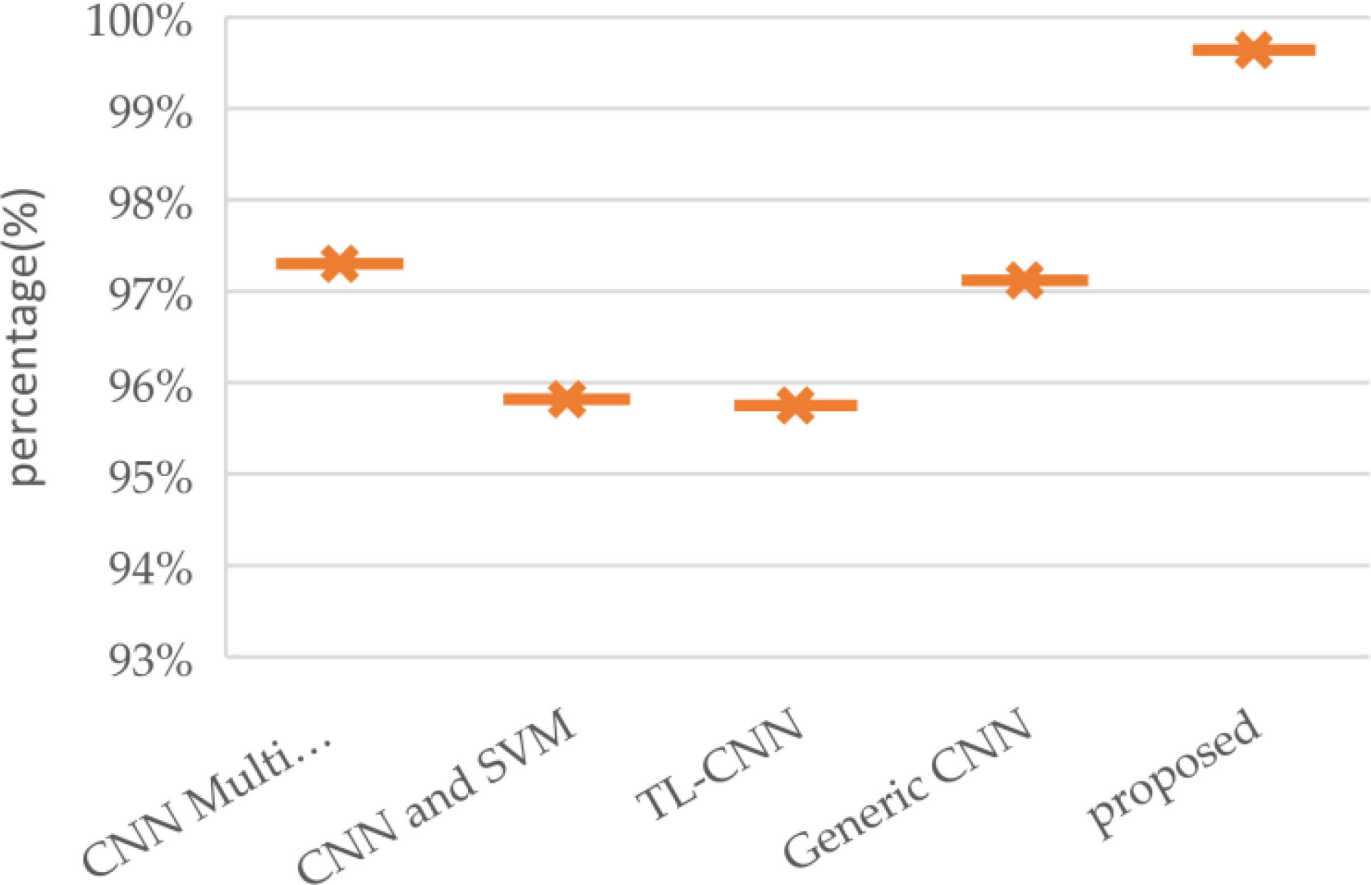
Comparison graph comparison of proposed method with Convolution Neural Network.

**Figure 11** provides a comparative overview of brain tumor classification accuracy achieved by various approaches, highlighting the superior performance of the proposed model. The analysis encompasses a range of techniques (32), including a multi-scale CNN, a CNN combined with Support Vector Machines, a transfer learning-based CNN, a generic CNN, and the proposed model. The performance metric accuracy, which is typically used in classification tasks, has been utilized to evaluate each approach to classifying. In this evaluation, the suggested model was able to achieve an impressive performance rate, demonstrating its capability to improve the classification accuracy of brain tumors. This is because it has been able to extract various distinctive and robust features from the images of the tumors. The suggested model was also able to distinguish different types of brain tumors. Its advanced structures were thoroughly tuned by using optimization techniques. In addition, the inclusion of more layers significantly improved its accuracy. The proposed models were able to improve their accuracy by incorporating more layers, which help them analyze medical images of brain tumors. The results of the evaluation revealed that the suggested model was able achieve a 99.64% precision rate. The high precision scores achieved by the two proposed models indicated their capability to properly categorize tumors.

## 4 Discussion

The goal is to create a vision transformer and a neural network that can be utilized to detect brain tumors utilizing MRI scans. Through the model, it was able to identify the different types of brain tumors, such as meningioma, pituitary, glioma, and no tumors. It demonstrated the potential of this type of vision transformer in this domain. Despite its accuracy, the study noted that the model could not guarantee a robust performance. To address this issue, it incorporated various metrics such as recall, precision, F1 score, and complexity analysis. A training and validation graph was also generated. The training accuracy of the model improved steadily over the course of the study. However, its validation accuracy started to show signs of overfitting. It eventually stabilized at 0.97. This shows that the model can generalize to unlabeled data. The study’s findings support the claim that the CNN-ViT model is capable of accurately detecting brain tumors. It also emphasizes the need for further evaluation and addressing issues related to overfitting.

A comprehensive classification evaluation of the CNN-ViT model has validated its capability to detect brain tumors. The model was able to detect glioma tumors with a precision of 0.91. This shows that it can identify this type of tumor with high accuracy. It was also able to identify about 82% of the actual glioma tumor cases with an F1 accuracy of 86%. In addition, the model was able to identify various types of tumors, such as meningiomas and pituitary tumors, with high accuracy and recall. It performed well in all three metrics for the non-tumor class. The results of these assessments reinforce the CNN-ViT classification system’s reliability and accuracy in identifying brain tumors.

The study emphasizes the importance of taking into account the various factors that affect the quality of data and the performance of the model when evaluating its results. **Table 4** compares the proposed classification model with the current techniques. This highlights its superior performance, and the inclusion of a healthy brain class also adds to its complexity. This is typically not considered in previous studies.

**Table 4:** Comparison with previous work.

Model	Dataset	Classes	Best Model	Accuracy
Nine pre-trained TL classifiers (37)	SARTAJ	3	InceptionResNetV2	98.91%
CNN and SVM (38)	Figshare	3	–	95.82%
TL-CNN (39)	Figshare, SARTAJ, BR35H	3	Developed TL CNN	95.75%
Generic CNN and six TL models (40)	Figshare, BR35H and SARTAJ combination	4	InceptionV3	97.12%
Proposed	Figshare, BR35H and SARTAJ combination	4	CNN	99.64

### 4.1 Limitations of the study

Training CNNs and ViTs on how to use high-quality datasets can be challenging due to the scarcity of data. This is because diverse sets of information are essential when creating models that generalize across different attributes. Transfer learning or data augmentation are usually needed when the data is limited. Another issue is domain adaptation, as trained models may not perform well on the other datasets. High-quality input data is also important for ViTs, as they rely on it for their attention-focused processing. Model interpretability is a must, as deep learning frameworks are opaque. Comprehending the classification process helps build trust in a model and provides insight into how each architecture handles images. This is important when evaluating CNNs or ViTs. When there are biases in a model or data, they can lead to the incorrect classification of an individual or group. This is why it is important that CNNs and ViTs take the necessary steps to reduce these biases. Compared to CNNs, ViTs are more computationally demanding, which may make them unsuitable for certain applications. This study faced limitations regarding dataset availability and computational complexity. Accessing comprehensive and current medical datasets for brain tumor classification remains a challenge due to privacy concerns and data scarcity. While public repositories like Kaggle offer valuable resources, obtaining diverse and large-scale datasets remains crucial for developing robust models. Additionally, the computational demands of training complex models like CNN-ViT necessitate significant processing power. Utilizing cloud-based platforms like Google Colab’s free tier can partially mitigate this, but researchers often face resource constraints and time limitations. Acquiring higher-tier services or dedicated high-performance computing resources would significantly expedite the research process, enabling more extensive experimentation and model optimization.

## 5 Conclusion

The study revealed that the ViT and CNN models performed well when it came to identifying brain tumors. The CNN models performed well on both tests and training data, while the ViT performed better on all parameters. It has been theorized that the ViT model overfitted with the data it was trained with, which is why it performed slightly below CNN’s classifiers. But, both of these can be utilized to identify brain tumors. The two models can still be utilized to classify brain tumors. However, the limitations of the study are that only the CNN- and ViT models were trained using powerful computational tools. In addition, the researchers did not examine other methods that can be used to classify brain tumors. The researchers only trained the CNN- and ViT-based models due to their hardware limitations. The researchers also did not look into other methods that can be utilized to categorize brain tumors in MRI scans. They only explored four types of brain tumors. After analyzing the CNN and ViT models’ results, the researchers will now look into how they can be improved and expand their training to include more types of brain tumors.

We need to conduct further tests and investigations to see if our suggestion has been effective. In medical imaging, the detection of brain tumors is a major area of research. We use various learning frameworks and models in our work, and there is still a lot to be done in this field. New systems can be developed to improve the precision of a diagnosis, which can help medical professionals and patients make informed decisions. Such advances in diagnostic technology can help improve the outcomes of patients and enhance the systems’ capabilities. The data collected for the study will be used to analyze the model’s capabilities in detecting various brain lesions. Though the present work marks the initial step toward establishing a system for detecting brain tumors, further research is required to enhance its capabilities.

In the future, we might use transformer-based structures to detect brain tumors. This approach marks a departure from CNN’s conventional methods, and it gives a new perspective. The main objective is to extract more information-rich features, which would enhance the model’s capabilities in distinguishing patterns. The concept of simplifying the network structure adds a dynamic dimension to the work being carried out in this field. It allows us to discover new ways to detect brain tumors.

## Data Availability

The original contributions presented in the study are included in the article/supplementary material. Further inquiries can be directed to the corresponding author.

## References

[1] Shah V Kochar P. Brain cancer: implication to disease, therapeutic strategies and tumor targeted drug delivery approaches. Recent patents anti-cancer Drug discovery. (2018) 13:70–85. doi: 10.2174/1574892812666171129142023 29189177

[2] Tandel GS Biswas M Kakde OG Tiwari A Suri HS Turk M. A review on a deep learning perspective in brain cancer classification. Cancers. (2019) 11:111. doi: 10.3390/cancers11010111 30669406 PMC6356431

[3] Lah TT Novak M Breznik B. Brain Malignancies: Glioblastoma and brain metastases. In: Seminars in cancer biology, vol. 60. Academic Press (2020). p. 262–73. 10.1016/j.semcancer.2019.10.010 31654711

[4] Louis DN Perry A Wesseling P Brat DJ Cree IA Figarella-Branger D. The 2021 WHO classification of tumors of the central nervous system: a summary. Neuro-oncology. (2021) 23:1231–51. doi: 10.1093/neuonc/noab106 PMC8328013 34185076

[5] Mellor NG Chung SA Graham ES Day BW Unsworth CP. Eliciting calcium transients with UV nanosecond laser stimulation in adult patient-derived glioblastoma brain cancer cells *in vitro* . J Neural Eng. (2023) 20:066026. doi: 10.1088/1741-2552/ad0e7d 37988746

[6] Kanumuri C Madhavi CR. A survey: Brain tumor detection using MRI image with deep learning techniques. Smart Sustain Approaches Optimizing Perform Wireless Networks: Real-time Applications. (2022) 18:125–38. doi: 10.1002/9781119682554.ch6

[7] Hollon TC Pandian B Adapa AR Urias E Save AV Khalsa SS. Near real-time intraoperative brain tumor diagnosis using stimulated Raman histology and deep neural networks. Nat Med. (2020) 26:52–8. doi: 10.1038/s41591-019-0715-9 PMC6960329 31907460

[8] Al-Zoghby AM Al-Awadly EMK Moawad A Yehia N Ebada AI. Dual deep cnn for tumor brain classification. Diagnostics. (2023) 13:2050. doi: 10.3390/diagnostics13122050 37370945 PMC10297724

[9] Sharma S. Artificial intelligence for fracture diagnosis in orthopedic X-rays: current developments and future potential. SICOT-J. (2023) 9:12. doi: 10.1051/sicotj/2023018 37409882 PMC10324466

[10] Javaid M Haleem A Singh RP Suman R Rab S. Significance of machine learning in healthcare: Features, pillars and applications. Int J Intelligent Networks. (2022) 3:58–73. doi: 10.1016/j.ijin.2022.05.002

[11] Yang Z Chen M Kazemimoghadam M Ma L Stojadinovic S Timmerman R. Deep-learning and radiomics ensemble classifier for false positive reduction in brain metastases segmentation. Phys Med Biol. (2022) 67:025004. doi: 10.1088/1361-6560/ac4667 PMC8858586 34952535

[12] Houssein EH Emam MM Ali AA Suganthan PN. Deep and machine learning techniques for medical imaging-based breast cancer: A comprehensive review. Expert Syst Applications. (2021) 167:114161. doi: 10.1016/j.eswa.2020.114161

[13] Zhang Q Xu Y Zhang J Tao D. Vitaev2: Vision transformer advanced by exploring inductive bias for image recognition and beyond. Int J Comput Vision. (2023) 131:1141–62. doi: 10.1007/s11263-022-01739-w

[14] Asiri AA Shaf A Ali T Pasha MA Aamir M Irfan M. Advancing brain tumor classification through fine-tuned vision transformers: A comparative study of pre-trained models. Sensors. (2023) 23:7913. doi: 10.3390/s23187913 37765970 PMC10535333

[15] Abdusalomov AB Mukhiddinov M Whangbo TK. Brain tumor detection based on deep learning approaches and magnetic resonance imaging. Cancers. (2023) 15:4172. doi: 10.3390/cancers15164172 37627200 PMC10453020

[16] Ahmad B Sun J You Q Palade V Mao Z. Brain tumor classification using a combination of variational autoencoders and generative adversarial networks. Biomedicines. (2022) 10:223. doi: 10.3390/biomedicines10020223 35203433 PMC8869455

[17] Talukder MA Islam MM Uddin MA Akhter A Pramanik MA Aryal S. An efficient deep learning model to categorize brain tumor using reconstruction and fine-tuning. Expert Syst applications. (2023) 230:120534. doi: 10.1016/j.eswa.2023.120534

[18] Polat Ö Güngen C. Classification of brain tumors from MR images using deep transfer learning. J Supercomputing. (2021) 77:7236–52. doi: 10.1007/s11227-020-03572-9

[19] Samee NA Mahmoud NF Atteia G Abdallah HA Alabdulhafith M Al-Gaashani MS. Classification framework for medical diagnosis of brain tumor with an effective hybrid transfer learning model. Diagnostics. (2022) 12:2541. doi: 10.3390/diagnostics12102541 36292230 PMC9600529

[20] Alanazi MF Ali MU Hussain SJ Zafar A Mohatram M Irfan M. Brain tumor/mass classification framework using magnetic-resonance-imaging-based isolated and developed transfer deep-learning model. Sensors. (2022) 22:372. doi: 10.3390/s22010372 35009911 PMC8749789

[21] Ullah N Khan JA Khan MS Khan W Hassan I Obayya M. An effective approach to detect and identify brain tumors using transfer learning. Appl Sci. (2022) 12:5645. doi: 10.3390/app12115645

[22] Ait Amou M Xia K Kamhi S Mouhafid M. A novel MRI diagnosis method for brain tumor classification based on CNN and Bayesian Optimization. InHealthcare. (2022) 10:494. doi: 10.3390/healthcare10030494 PMC8949584 35326972

[23] Saeedi S Rezayi S Keshavarz H R. Niakan Kalhori S. MRI-based brain tumor detection using convolutional deep learning methods and chosen machine learning techniques. BMC Med Inf Decision Making. (2023) 23:16. doi: 10.1186/s12911-023-02114-6 PMC9872362 36691030

[24] Huang Z Du X Chen L Li Y Liu M Chou Y. Convolutional neural network based on complex networks for brain tumor image classification with a modified activation function. IEEE Access. (2020) 8:89281–90. doi: 10.1109/Access.6287639

[25] Asiri AA Aamir M Shaf A Ali T Zeeshan M Irfan M. Block-wise neural network for brain tumor identification in magnetic resonance images. Computers Materials Continua. (2022) 73:5735–5753. doi: 10.32604/cmc.2022.031747

[26] Muezzinoglu T Baygin N Tuncer I Barua PD Baygin M Dogan S. PatchResNet: multiple patch division–based deep feature fusion framework for brain tumor classification using MRI images. J Digital Imaging. (2023) 36:973–87. doi: 10.1007/s10278-023-00789-x PMC10287865 36797543

[27] Asiri AA Shaf A Ali T Aamir M Usman A Irfan M. Multi-level deep generative adversarial networks for brain tumor classification on magnetic resonance images. Intelligent Automation Soft Computing. (2023) 36:127–143. doi: 10.32604/iasc.2023.032391

[28] Asiri AA Shaf A Ali T Shakeel U Irfan M Mehdar KM. Exploring the power of deep learning: fine-tuned vision transformer for accurate and efficient brain tumor detection in MRI scans. Diagnostics. (2023) 13:2094. doi: 10.3390/diagnostics13122094 37370989 PMC10297056

[29] Asiri AA Shaf A Ali T Pasha MA Aamir M Irfan M. Advancing brain tumor classification through fine-tuned vision transformers: A comparative study of pre-trained models. Sensors. (2023) 23:7913. doi: 10.3390/s23187913 37765970 PMC10535333

[30] Deepak S Ameer PM. Brain tumor classification using deep CNN features via transfer learning. Comput Biol Med. (2019) 111:103345. doi: 10.1016/j.compbiomed.2019.103345 31279167

[31] Akter A Nosheen N Ahmed S Hossain M Yousuf MA Almoyad MA. Robust clinical applicable CNN and U-Net based algorithm for MRI classification and segmentation for brain tumor. Expert Syst Applications. (2024) 238:122347. doi: 10.1016/j.eswa.2023.122347

[32] Gómez-Guzmán MA Jiménez-Beristaín L García-Guerrero EE López-Bonilla OR Tamayo-Perez UJ Esqueda-Elizondo JJ. Classifying brain tumors on magnetic resonance imaging by using convolutional neural networks. Electronics. (2023) 12:955. doi: 10.3390/electronics12040955

[33] Kaggle brain tumor MRI dataset. Available online at: 10.34740/KAGGLE/DSV/2645886 (Accessed 10 September 2022).

[34] Brain tumor MRI dataset . Available online at: https://figshare.com/articles/dataset/brain_tumor_dataset/1512427 (Accessed 10 October 2022).

[35] Kaggle brain tumor MRI dataset. Available online at: 10.34740/KAGGLE/DSV/1183165 (Accessed 10 September 2022).

[36] Kaggle brain tumor MRI dataset. Available online at: https://www.kaggle.com/datasets/ahmedhamada0/brain-tumor-detection?select=no (Accessed 10 September 2022).

[37] Ullah N Khan J Khan MS Khan W Hassan I Obayya M. An effective approach to detect and identify brain tumors using transfer learning. Appl Sci. (2022) 12:5645. doi: 10.3390/app12115645

[38] Deepak S Ameer PM. Automated categorization of brain tumor from mri using cnn features and svm. J Ambient Intell Humaniz.Comput. (2021) 12:8357–69. doi: 10.1007/s12652-020-02568-w

[39] Alanazi MF Ali MU Hussain SJ Zafar A Mohatram M Irfan M. Brain tumor/mass classification framework using magnetic-resonance-imaging-based isolated and developed transfer deep-learning model. Sensors. (2022) 22:372. doi: 10.3390/s22010372 35009911 PMC8749789

[40] Gómez-Guzmán MA Jiménez-Beristaín L García-Guerrero EE López-Bonilla OR Tamayo-Perez UJ Esqueda-Elizondo JJ. Classifying brain tumors on magnetic resonance imaging by using convolutional neural networks. Electron Electron. (2023) 12(4):955. doi: 10.3390/electronics12040955

[41] Bonada M Rossi LF Carone G Panico F Cofano F Fiaschi P. Deep Learning for MRI segmentation and molecular subtyping in glioblastoma: critical aspects from an emerging field. Biomedicines. (2024) 12:p.1878. doi: 10.3390/biomedicines12081878 PMC11352020 39200342

[42] Yahyaoui H Ghazouani F Farah IR. July. Deep learning guided by an ontology for medical images classification using a multimodal fusion. In: 2021 international congress of advanced technology and engineering (ICOTEN). Taiz, Yemen: IEEE (2021). p. 1–6.

[43] Khan AR Khan S Harouni M Abbasi R Iqbal S Mehmood Z. Brain tumor segmentation using K-means clustering and deep learning with synthetic data augmentation for classification. Microscopy Res Technique. (2021) 84:1389–99. doi: 10.1002/jemt.23694 33524220

[44] Latif G Ben Brahim G Iskandar DA Bashar A Alghazo J. Glioma Tumors’ classification using deep-neural-network-based features with SVM classifier. Diagnostics. (2022) 12:1018. doi: 10.3390/diagnostics12041018 35454066 PMC9032951

[45] Bhatele KR Bhadauria SS. Machine learning application in glioma classification: review and comparison analysis. Arch Comput Methods Eng. (2022) 29:247–74. doi: 10.1007/s11831-021-09572-z

[46] Murthy MYB Koteswararao A Babu MS. Adaptive fuzzy deformable fusion and optimized CNN with ensemble classification for automated brain tumor diagnosis. Biomed Eng Lett. (2022) 12:37–58. doi: 10.1007/s13534-021-00209-5 35186359 PMC8825897

